# Host mixtures for plant disease control: Benefits from pathogen selection and immune priming

**DOI:** 10.1111/eva.13386

**Published:** 2022-05-23

**Authors:** Pauline Clin, Frédéric Grognard, Didier Andrivon, Ludovic Mailleret, Frédéric M. Hamelin

**Affiliations:** ^1^ Institut Agro, INRAE, IGEPP Univ Rennes Rennes France; ^2^ INRAE, CNRS, ISA Université Côte d’Azur Nice France; ^3^ Inria, INRAE, CNRS, Sorbonne Université, Biocore Université Côte d’Azur Nice France

**Keywords:** durable management of resistance, gene‐for‐gene interaction, induced resistance, sustainable agriculture, systemic acquired resistance, virulence

## Abstract

Multiline and cultivar mixtures are highly effective methods for agroecological plant disease control. Priming‐induced cross protection, occurring when plants are challenged by avirulent pathogen genotypes and resulting in increased resistance to subsequent infection by virulent ones, is one critical key to their lasting performance against polymorphic pathogen populations. Strikingly, this mechanism was until recently absent from mathematical models aiming at designing optimal host mixtures. We developed an epidemiological model to explore the effect of host mixtures composed of variable numbers of single‐resistance cultivars on the equilibrium prevalence of the disease caused by pathogen populations polymorphic for virulence complexity. This model shows that a relatively large amount of resistance genes must be deployed to achieve low disease prevalence, as pathogen competition in mixtures tends to select for intermediate virulence complexity. By contrast, priming significantly reduces the number of plant genotypes needed to drop disease prevalence below an acceptable threshold. Given the limited availability of resistance genes in cultivars, this mechanism of plant immunity should be assessed when designing host mixtures.

## 1 INTRODUCTION

It has long been recognized that sustainable agriculture requires profound changes in agricultural practices (Eyhorn et al., 2019). Still, the vast majority of agro‐ecosystems are grown as monocultures, that is, as large and continuous deployment of a single plant genotype over potentially vast areas (McDonald & Stukenbrock, 2016). The development of mechanization, plant breeding, and crop protection products promotes the use of monocultures because they facilitate crop management and ensure high yields under current management practices (Wuest et al., 2021).

Unfortunately, these homogeneous and simplified agricultural landscapes are particularly vulnerable to disease outbreaks (Brown, 2015), since pathogens are best transmitted in genetically similar environments (Stukenbrock & McDonald, 2008). In addition, genetically identical crops increase the selection pressure toward pathogens overcoming plant resistance. As a result, defeated resistances are no longer of agronomic value, the available genetic diversity is eroding (Thrupp, 2000), and the need for pesticides increases (Brown & Tellier, 2011).

Sustainable agriculture requires growers to preserve diversity and reduce inputs within the crops (Renard & Tilman, 2021). In addition to adequately managing the available genetic resources, increasing the diversity used allows growers both to maintain yields (Brooker et al., 2015; Prieto et al., 2015; Wuest et al., 2021) and to prevent the emergence of new pathogens (McDonald & Stukenbrock, 2016).

Several methods exist to increase plant diversity at the field scale. One is the use of intercropping, which consists of mixing several crop species at the same time and in the same field, thus increasing interspecific diversity (Boudreau, 2013). Another is to increase intraspecific plant diversity through cultivar or multiline mixtures (Mundt, 2002; Reiss & Drinkwater, 2018).

Cultivar mixtures consist in cultivating several cultivars of the same species but with different agronomic traits (including disease resistance) at the same time and in the same field (Wolfe, 1985). Their efficiency has been demonstrated for several decades across different pathosystems (Kristoffersen et al., 2020; Mundt, 2002; Reiss & Drinkwater, 2018; Yang et al., 2019). However, mixtures generally contain cultivars that may have different phenotypes in terms of plant size or harvest date for example. Under current agricultural practices, these differences require adaptation and may have a significant management cost, which compromise the adoption of cultivar mixtures (Brooker et al., 2015).

Multiline mixtures, by contrast, are constructed by mixing lines selected for phenotypic homogeneity of agronomic traits, except disease resistance (Browning & Frey, 1969). More specifically, multilines are near‐isogenic lines differing only in the presence or absence of resistance genes against a specific disease (Mundt, 2002). Multilines have been widely grown over large areas in several parts of the world for decades (de Vallavieille‐Pope, 2004), for instance in the United States against wheat stripe rust (Chen, 2007), in Japan against rice blast (Fukuoka & Okuno, 2019), and in Columbia against coffee leaf rust (Avelino et al., 2015). The number of varieties (or resistance genes) in the multilines is variable: 10 in Crew and Rely wheat cultivars (Allan et al., 1983, 1993), 2 to 4 (among 11) in Koshihikari BL and Sasanishiki BL rice cultivars (Abe, 2004; Ishizaki et al., 2005), and 5 to 11 in Castillo and Colombia coffee cultivars (Romero et al., 2014; Ward et al., 2017). The agronomic homogeneity conferred by multilines is compatible with current agricultural practices, which facilitates their use (Finckh et al., 2000).

Although multilines are often selected against a single disease, their genetic diversity may trigger immune priming, thereby protecting the crop against other diseases as well (Finckh et al., 2000). More specifically, pathogen genotypes that cannot circumvent a resistance gene often trigger resistance priming in the host. Priming confers an increased level of immunity against a large spectrum of pathogens (Wilkinson et al., 2019) including the targeted one. Actually, priming is known as a key element of the success of host mixtures, both in practice (e.g., Calonnec et al., 1996; Lannou et al., 2005) and in theory (Clin et al., 2021; Lannou et al., 1995). For instance, priming is considered to decrease disease prevalence in multilines of cereal crops, for example, rice against blast (Koizumi et al., 2004).

An initial fear concerning multilines was the selection for “complex” pathogen genotypes which would circumvent all or nearly all resistance genes in the mixture (Carson, 2009; Mundt, 2014). The number of resistance genes a pathogen is able to overcome is termed “virulence complexity” in plant pathology (Milgroom, 2015). However, both modeling (Lannou & Mundt, 1997; Xu, 2012) and empirical evidence (Dileone & Mundt, 1994; Lannou et al., 2005; Leung et al., 2003) suggest that this risk might actually be limited. This is in line with early mathematical models on the subject (reviewed in Kiyosawa, 1982; Leonard & Czochor, 1980), which show that evolution yields convergence toward an intermediate virulence complexity, provided there is a cost to resistance‐breaking (Groth, 1976). However, these were mainly population genetic models tracking pathogen genotype frequencies, but ignoring population densities. Therefore, these models were not designed to fully assess the epidemiological efficiency of multiline mixtures.

Specifically, the questions we ask are as follows: (i) how does the prevalence of the disease depend on the number of resistant lines in the mixture? (ii) what is the additional effect of priming on this relationship? To address these questions, we built an epidemiological model allowing us to understand the dynamics of the pathogen population accounting for all possible host–pathogen interactions in multiline mixtures. By analyzing our model analytically, we first determine the existence of a diversity threshold, that is, the necessary but sufficient number of components in the mixture to eradicate a pathogen in the absence of priming. We also evaluate the additional effect of immune priming on disease reduction within mixtures.

## 2 MODELING

Let us consider a multiline of *n* resistant lines, each having a single, but different, race‐resistance gene, R*_i_*, with *i* = 1,…,*n*. Table 1 shows an example of a gene‐for‐gene interaction matrix, following Flor (1971)’s gene‐for‐gene model and taking priming into account for *n* = 3.

**TABLE 1:** Host–pathogen interactions in a multiline composed of 3 resistant lines

Pathogen	av_1_/Av_2_/Av_3_	Av_1_/av_2_/Av_3_	Av_1_/Av_2_/av_3_	av_1_/av_2_/Av_3_	av_1_/Av_2_/av_3_	Av_1_/av_2_/av_3_	av_1_/av_2_/av_3_
Line							
*R*_1_	+	*	*	+	+	*	+
*R*_2_	*	+	*	+	*	+	+
*R*_3_	*	*	+	*	+	+	+
Virulence complexity	1	1	1	2	2	2	3

Notes

Each resistant line (row) corresponds to a single resistance gene (either *R*_1_, *R*_2_ or *R*_3_). There are seven possible pathogen genotypes that are able to infect at least one component of the multiline (columns): av_1_/Av_2_/Av_3_, Av_1_/av_2_/Av_3_,…, av_1_/av_2_/av_3_ (Av means “avirulent” and av means “virulent”). For instance, av_1_/av_2_/av_3_ means that this pathogen genotype is able to infect *R*_1_, *R*_2_, and *R*_3_: this is a triply virulent pathogen genotype. In contrast, av_1_/av_2_/Av_3_, av_1_/Av_2_/av_3_, Av_1_/av_2_/av_3_ cannot infect *R*_3_, *R*_2_ and *R*_1_, respectively, but instead trigger immune priming on *R*_3_, *R*_2_ and *R*_1_, respectively. They are doubly virulent. Singly virulent pathogen genotypes, av_1_/Av_2_/Av_3_, Av_1_/av_2_/Av_3_, and Av_1_/Av_2_/av_3_, are able to infect *R*_1_, *R*_2_ and *R*_3_, respectively, and trigger priming on *R*_2_ and *R*_3_, *R*_1_ and *R*_3_, and *R*_2_ and *R*_1_, respectively. We ignore triply avirulent (Av_1_/Av_2_/Av_3_) pathogen genotypes since they cannot infect any resistant variety and therefore cannot invade host mixtures considered in this study. Virulence complexity is the number of varieties a pathogen genotype can infect.

+, infection; ∗, priming.

The total host density in the mixture is a constant. All varieties are present in the same proportion and are assumed to be equal from an epidemiological standpoint. For instance, the maximum possible pathogen transmission rate *R* is the same for all varieties. These assumptions best fit multiline mixtures, in which the resistant varieties are often used in equal proportions, as in for example, the Rely cultivar of wheat against stripe rust (Chen, 2007).

Bearing a virulence gene (av*_i_*) involves a cost *c* to the pathogen, reducing its transmission rate by a factor 0 ≤ 1 – c ≤ 1, as compared to an avirulent genotype on a variety with no resistance gene. The virulence cost is the same for all virulence genes. The idea of a cost as a counterpart of the ability of breaking a resistance gene originated as a theoretical hypothesis to explain the often‐observed maintenance of polymorphism in pathogen populations, in both agricultural and wild ecosystems (Brown, 2015; Gandon et al., 2002; Sasaki, 2000; Tellier & Brown, 2007b; Vanderplank, 1968). Since then, such a cost has been demonstrated and measured in a number of parasites, including bacteria (Cruz et al., 2000; Wichmann & Bergelson, 2004), fungi (Bahri et al., 2009; Bousset et al., 2018; Bruns et al., 2014; Caffier et al., 2010; Carson, 1998; Huang et al., 2010; Thrall & Burdon, 2003), viruses (Fraile et al., 2010; Ishibashi et al., 2012; Janzac et al., 2010; Jenner et al., 2002; Khatabi et al., 2013; Poulicard et al., 2010), nematodes (Castagnone‐Sereno et al., 2007), and oomycetes (Montarry et al., 2010).

The virulence cost is assumed to be multiplicative, meaning that if a pathogen genotype has *k* virulence genes, it bears a fitness cost (1 − *c*)*^k^* as compared to an avirulent genotype on a variety with no resistance gene (Groth, 1976; Kiyosawa, 1982; Leonard & Czochor, 1980; Marshall & Pryor, 1978; Ostergaard, 1983; Sasaki, 2000; Segarra, 2005; Tellier & Brown, 2007a). We denote as *k* the virulence complexity of a pathogen genotype, that is, the number of virulence genes it possesses. Pathogen genotypes of virulence complexity *k* have a net transmission rate *R*(1−*c*)*^k^* from the varieties they can infect.

The priming efficiency, 0 ≤ *ρ* ≤ 1, reduces the probability that a host is infected by a virulent pathogen genotype. Experimental evidence suggests that priming usually becomes effective a few hours or days after challenge with an avirulent pathogen genotypes (Maleck et al., 2000; Ross, 1961). However, we ignore this delay for simplicity. Note that priming can be fully effective (Kuć, 1982). In such a case (*ρ* = 1), a virulent pathogen genotype cannot infect a primed host as long as priming is active.

The rate at which priming loses its efficiency is *γ*. It corresponds to the inverse of the mean time during which priming is effective. Several studies have shown that priming can last for several weeks. The original one (Ross, 1961) estimates that it persists for 20 days, but more recent reports show that it can last for weeks to months (Fu & Dong, 2013; Kuć, 1982).

We consider a continuous‐time model with continuous planting and replanting best adapted to perennial crops in tropical regions (Madden et al., 2007). More specifically, we consider that the host is present year long and we ignore seasonality in climatic conditions for simplicity. This will allow us to identify the general mechanisms promoting the success (or failure) of host mixtures, which are expected to hold in annual crops as well.

Infected hosts remain infectious until harvest, as is the case for most plant viruses and many other parasites. The rate at which a host is replaced with an uninfected one (due to harvesting and replanting) is *α*. It corresponds to the inverse of the length of the growing season.

Since the varieties are epidemiologically equal, they are interchangeable, and it is sufficient to keep track of the disease on a single variety to model the full epidemic (Section S1). Therefore, we focus on one variety among the *n* varieties present in the mixture, hereafter called the focal variety. We denote by *x_k_* the density of hosts of the focal variety that are infected by a given pathogen genotype of virulence complexity *k* = 1, …, *n*. The infection force of a given pathogen genotype having virulence complexity *k* is $f_{k}=\text{kR}{\left(1-c\right)}^{k}x_{k}$ since its per capita transmission rate is *R*(1 − *c*)*^k^* and its density is *kx_k_*. The density of hosts of the focal variety that are primed is *m*. The density of uninfected and unprimed hosts for the focal variety is defined as *X*. The priming force is *P*. It is proportional to the density of pathogen genotypes that can trigger priming on the focal variety. The infection force of the pathogen population on the focal variety is *F*. It is proportional to the density of pathogen genotypes that can infect the focal variety. The expressions for *X*,*P*, and *F* are provided in Supplementary Information (Eq. S8, S9, and S10, respectively).

Without loss of generality, we take the average length of the growing season (1/*α*) as a unit of time. We introduce the re‐scaled removal rate ν = (*α* + *γ*)/*α*, meaning that primed hosts can be removed due to either harvest or loss of priming.

The model is expressed as a system of *n* + 1 ordinary differential equations, in which the prime denotes differentiation with respect to time: for *k* = 1, …, *n*,

(1) $$\begin{array}{rcl} \underset{\begin{array}{c} \text{rate of change of hosts}\\ \text{of the focal variety}\\ \text{infected by a genotype}\\ \text{of complexity }k \end{array}}{\underbrace{x_{k}^{\prime }}} & = & \underset{\begin{array}{c} \text{infection force}\\ \text{of a genotype}\\ \text{of complexity }k \end{array}}{\underbrace{f_{k}}}(\underset{\begin{array}{c} \text{uninfected and}\\ \text{unprimed hosts}\\ \text{of the focal variety} \end{array}}{\underbrace{X}}+\;(1-\rho )\underset{\begin{array}{c} \text{hosts of the}\\ \text{focal variety}\\ \text{that are primed} \end{array}}{\underbrace{m}})\;-\underset{\begin{array}{c} \text{harvested}\\ \text{hosts} \end{array}}{\underbrace{x_{k}}}\;,\\ \underset{\begin{array}{c} \text{rate of change of hosts}\\ \text{of the focal variety}\\ \text{that are primed} \end{array}}{\underbrace{m^{\prime }}} & = & \underset{\begin{array}{c} \text{priming force}\\ \text{of the pathogen}\\ \text{population} \end{array}}{\underbrace{P}}X\;-\underset{\begin{array}{c} \text{infection force of}\\ \text{the pathogen}\\ \text{population} \end{array}}{\underbrace{F}}(1-\rho )m\;-\underset{\begin{array}{c} \text{loss of}\\ \text{priming}\\ \text{or harvest} \end{array}}{\underbrace{\nu m}}\;. \end{array}$$

The analysis of the model is provided in Supplementary Information (Section S3). We assumed that the maximum pathogen diversity was initially present (i.e., $2^{n}-1$ pathogen genotypes, e.g., 31 for *n* = 5, or 1023 for *n* = 10), and we let the host‐pathogen dynamics determine which pathogen genotypes go extinct. The model notations and their definitions are listed in Table 2.

**TABLE 2:** Model parameters and variables

	Definition
Parameter	
*n*	Number of resistant varieties in the mixture
R_i_	Variety with a single resistance gene at locus *i* = 1, …, *n*
*k*	Virulence complexity of pathogen genotype: *k* = 1, …, *n*
*c*	Virulence cost: c∈[0,1]
*ρ*	Priming effect: ρ∈[0,1]
*γ*	Priming loss rate: *γ* ≥ 0
*α*	Harvest and replanting rate: *α* > 0
*R*	Transmission rate: *R* > 1
*ν*	Re‐scaled removal rate: ν=(γ+α)/α≥1
Variable	
*x_k_*	Proportion of hosts of the focal variety infected by a pathogen of virulence complexity *k*
*m*	Proportion of primed hosts for the focal variety
*X*	Proportion of uninfected and unprimed hosts for the focal variety

## 3 RESULTS

### 3.1 Intraspecific competition selects for an intermediate virulence complexity

**FIGURE 1:**
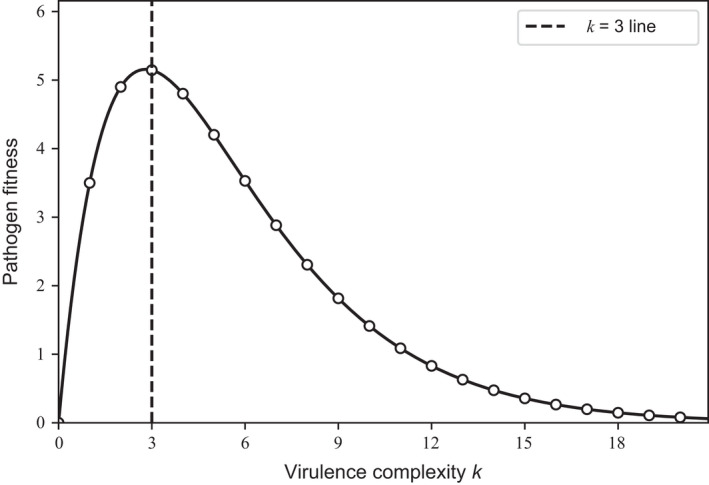
Pathogen fitness, $\phi_{k}=kR{\left(1-c\right)}^{k}$, as a function of virulence complexity *k*. Virulence complexity is the number of varieties a pathogen can infect, subject to a multiplicative cost *c*. The maximum possible transmission rate is *R*. The pathogen fitness is maximized for an intermediate level of virulence complexity, *k* ^*^. For the parameter values taken in this example (*R* = 5 and *c* = 0.3), $k^{*}=3$. Note that the dotted line does not go through the maximum of the curve because *k* can take only integer values

The analysis of our model revealed that only one virulence complexity, *k* ^*^, persists in the pathogen population at endemic equilibrium (Supplementary Information, Theorem S1). This virulence complexity, *k* ^*^, turns out to be the one that maximizes $\phi_{k}=kR{\left(1-c\right)}^{k}$, which emerges as a metric of fitness. Figure 1 shows that pathogen fitness, $\phi_{k}$, is maximized for an intermediate level of virulence complexity, *k* ^*^.

As a result, the number of genotypes in the pathogen population is the number of possible virulence combinations resulting in pathogens of virulence complexity *k* ^*^, that is $\left(\begin{array}{c} n\\ k^{*} \end{array}\right)$ (e.g., 10 for *n* = 5 and *k* ^*^ = 3, or 120 for *n* = 10 and *k* ^*^ = 3).

### 3.2 How many varieties are needed to eradicate the disease?

**FIGURE 2:**
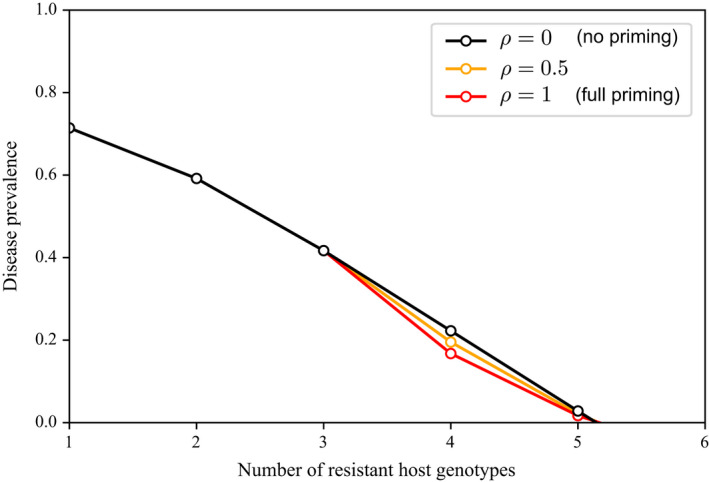
Total prevalence of the disease at equilibrium, $\bar{P}$, as a function of the number of varieties in the mixture *n*. Parameter values: *R* = 5 (transmission rate), *c* = 0.3 (virulence cost), and *ν* = 1 (re‐scaled removal rate). In this case, using 5 varieties in the mixture is sufficient to eradicate the disease. The priming efficiency (*ρ*) has little influence on the prevalence

Figure 2 shows that it is possible to eradicate the disease provided a sufficient number of varieties is present in the mixture. The minimum number of varieties required to eradicate the disease can be approximated as $n_{c}=R/\left(‐\log\left(1‐c\right)e\right)$, where *e* = exp(1). For the parameter set used in Figure 2, $n_{c}\approx 5.15$. This approximation holds regardless whether priming occurs (*ρ* > 0) or not (*ρ* = 0). However, for *n_c_* to keep a reasonably low value, *R* must be relatively low, and *c* must not be too low (Section S5). For instance, Figure 2 shows that mixing $n_{c}\approx 5$ varieties enables eradicating the disease for *R* = 5 and *c* = 0.3.

### 3.3 Priming may strongly reduce the prevalence of the disease

**FIGURE 3:**
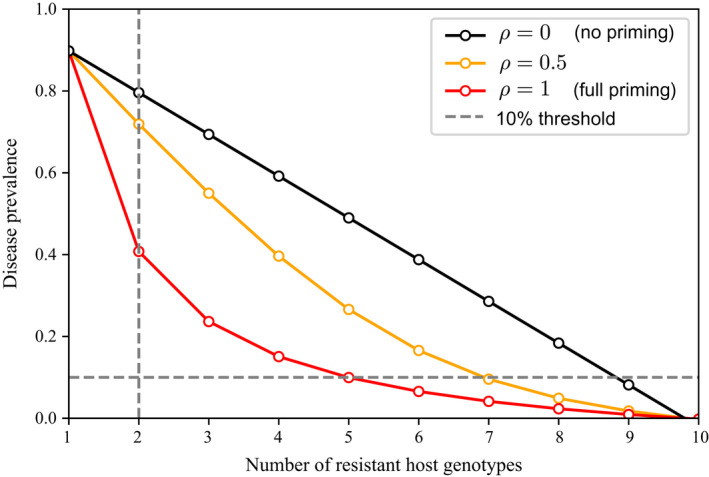
Total equilibrium prevalence of the disease, $\bar{P}$, as a function of the number of varieties in the mixture *n*. The 10% prevalence threshold corresponds to a possible acceptable threshold in an agroecological context. Parameters values: *R* = 20 (transmission rate), *c* = 0.5 (virulence cost) and *ν* = 1 (re‐scaled removal rate). In this case, the number of varieties needed to bring the prevalence below the 10% threshold can be reduced from 9 to 5 through priming. The *n* = 2 line corresponds to a possible situation in which the genetic resource is limited to *n* = 2. The prevalence can be reduced from 0.8 to 0.4 through priming

Because the genetic resource is limited, it may not be possible to eradicate the disease. For instance, Figure 3 shows that, for *R* = 20 and *c* = 0.5, a relatively high number of varieties would be needed to eradicate the disease ($n_{c}\approx 10$). Nevertheless, in an agroecological context, the objective may be not to exceed a prevalence threshold of 10% for example. The number of varieties required to achieve this objective depends on whether priming is effective or not. Figure 3 shows that the number of varieties needed to bring the prevalence below the 10% threshold can be reduced from nine to five through priming. Moreover, even if less than five varieties are available, it is still possible to strongly reduce the prevalence through priming. For instance, Figure 3 shows a possible situation in which mixture involves only two varieties: Then, disease prevalence can be reduced from 0.8 to 0.4 through priming.

Additional figures showing qualitatively similar results with other parameter sets can be found in Supplementary Information (Figures S7 and S8). Our work is also supported by an interactive interface allowing the user to test their own parameter sets: https://share.streamlit.io/paulineclin/multiresistance_priming_model/main/app.py.

## 4 DISCUSSION

Bio‐diversification is one leading principle of agroecology (Altieri, 2018). Host diversification methods include cultivar and multiline mixtures. The abundant research on mixtures efficacy includes a number of modeling and simulation studies, recently reviewed in Rimbaud et al. (2021). However, few simple epidemiological models have been developed to mathematically analyze host mixtures, unravel their mechanisms, and optimize their design (e.g., Clin et al., 2021; Mikaberidze et al., 2015). Most early models (reviewed in Kiyosawa, 1982; Leonard & Czochor, 1980) were population genetics models targeting pathogen evolution. They were therefore not designed to answer questions such as the number of varieties needed to eradicate a disease or the effect of priming on disease prevalence. In this study, we developed a model to address these questions both for cultivar and multiline mixtures mixing qualitative resistance genes.

Our study showed that intraspecific competition in the pathogen population selects for an intermediate virulence complexity (Figure 1). This result is akin to competitive exclusion in ecology. The pathogen population is however composed of a set of genotypes having the same virulence complexity. The virulence complexity that is selected for is the one maximizing a metric of pathogen fitness. This metric of fitness is the same as in early population genetic models (e.g., Groth, 1976). However, these population genetic models (reviewed in Kiyosawa, 1982; Leonard & Czochor, 1980) assumed such a fitness metric, while ours emerges from the analysis of a novel epidemiological model. If the virulence cost is sufficiently large, an intermediate virulence complexity is selected for, meaning that evolution does not ultimately select for pathogen genotypes capable of breaking all the resistance genes in the mixture. However, we assumed that pathogen reproduction was clonal. Sexual reproduction and recombination, by regenerating some variability in virulence complexity, would challenge the observation of a unique virulence complexity in the pathogen population (McDonald & Linde, 2002). Nevertheless, the average virulence complexity might remain close to the one predicted by our asexual model.

Our epidemiological approach allowed us to show that it is possible to eradicate the disease provided a sufficient number of varieties is present in the mixture (Figure 2). From a different perspective, based on a model allowing for quantitative resistances, Mikaberidze et al. (2015) showed a similar result for fully or highly specialized pathogens. In our framework, fully or highly specialized pathogens correspond to singly virulent pathogens. We showed that disease eradication is still possible, in theory, when the pathogen population includes multivirulent genotypes, provided that a sufficient number of resistance genes are available. We additionally showed that in case the available number of varieties does not permit us to eradicate the disease, priming may nevertheless reduce the final prevalence of the disease (Figure 3). However, for priming to have a strong effect, the pathogen must have a large transmission rate (Mikaberidze et al., 2016), and the virulence cost must be relatively large as well (Nilusmas et al., 2020). This is because a high virulence cost selects for low virulence complexity, which maximizes host–pathogen interactions that trigger priming. In this way, priming increases the cross‐protection between varieties and consequently improves the durability of resistances.

In agroecology, the objective is often to decrease the prevalence of the disease below an acceptable threshold, instead of trying to eradicate the disease entirely. Again, if the transmission rate and the virulence cost are large, priming enables us to strongly decrease the number of varieties required to satisfy the acceptable threshold. For instance, Figure 3 shows that the number of varieties can be divided by half, which would make the use of host mixtures more achievable in practice. These results highlight the key role of priming in designing methods for sustainable agriculture.

Although tailored for multilines, we expect our model to apply to cultivar mixtures as well, provided the components of the mixture are in equal proportion, and each carries a single and specific resistance gene. However, cultivars in mixtures may contain multiple resistance genes (Koizumi, 2001). Therefore, extending the model to account for quantitative resistances or gene pyramiding would be a logical continuation of the study. Technically, this would consist in relaxing the assumptions that all hosts confer the same transmission rate to the pathogen, and that resistances are associated with the same virulence cost. Such extensions would likely challenge mathematical tractability, unless the number of varieties in the mixtures is reduced to a small number. This is a work under progress. From a broader perspective, our model shares several features (host heterogeneity, multiplicative costs, equal frequency of resistant host genotypes) with non‐plant models, including bacteriophages for instance (Chabas et al., 2018).

To go further in the study of host mixtures, it would be relevant to consider a susceptible variety in the mixture, as in for example (Kiyosawa, 1977). Susceptible varieties would form a refuge for an avirulent pathogen genotype, which would in turn trigger priming on the resistant varieties. Also, susceptible varieties are sometimes more economically profitable than resistant varieties (Ben M’Barek et al., 2020; Zhu et al., 2000), which would increase the expected benefits from mixtures. This extension of the present work is left for a future paper.

Our results should not be interpreted at a larger spatial scale, namely that of a landscape composed of a mosaic of monocultures with distinct resistance genes. This is because priming does not operate at the scale of a field. Other spatial aspects, including the spreading speeds of different pathogen genotypes in host mixtures, would however deserve to be investigated to better comprehend the multiple facets of host mixtures. Finally, the exploration of the stochastic and evolutionary dynamics of pathogens in host mixtures (Bourget et al., 2013; Chabas et al., 2018) could also complement or challenge some of the results of this article, concerning for example the number of varieties necessary to prevent pathogen invasion.

## 5 CONFLICT OF INTEREST

None declared.

## Supporting information

Supinfo S1

## Data Availability

Data sharing is not applicable to this article as no new data were created or analyzed in this study. The source code for our interactive online interface is available at https://github.com/PaulineClin/Multiresistance_priming_model.
