# Supplementary material for: Host mixtures for plant disease control: Benefits from pathogen selection and immune priming
Source: Evol Appl. 2022 May 23;15(6):967–75. doi: 10.1111/eva.13386 (PMC9234633; doi:10.1111/eva.13386)
Supplement: Supplementary file 1 — Supinfo S1 [file EVA-15-967-s001.pdf]

Supporting information for “Host mixtures for plant disease control: benefits from pathogen selection and immune priming”.

Pauline Clin, Frédéric Grognard, Didier Andrivon, Ludovic Mailleret, Frédéric M. Hamelin

Organisation of this document

This document consists of six sections:

- **S1. Lower dimension models.** This section introduces the full model for host mixtures containing a low number of resistant varieties ($n = 2$, and then $n = 3$). The complexity of the full model increases quickly with n. Specifically, the model is composed of $n(1 + 2^{n-1})$ coupled ordinary differential equations (e.g. 15 for $n = 3$). Therefore, we propose a way to reduce the model dimension based on a symmetry assumption. The reduced model has dimension $n + 1$.

- **S2. Model for an arbitrary number n of varieties.** This section shows how the reduced models from the previous section can be generalized to a mixture with n varieties. A biological interpretation of the different terms of the model is provided.

- **S3. Analysis.** This section shows model equilibria, whether they are biologically feasible, and whether they are locally stable. In particular, we show that there is a competitive exclusion principle whereby a single virulence complexity persists in the system.

- **S4. Warning about cases in which $1/c$ is an integer.** This section shows that there exist special cases in which the competitive exclusion principle and/or the symmetry assumption made to reduce the model dimension are not valid. However, these special cases are non-generic, i.e. biologically irrelevant. Therefore, their study is out of the scope of this paper.

- **S5. Prevalence of the disease**. This section shows how to compute the prevalence of the disease as a function of the number of varieties in the mixture, and how it varies with the model parameters.

- **S6. Area Under Disease Progress Curve (AUDPC)**. This section introduces the AUDPC as a metric of the epidemic size over time and shows how it depends on the number of varieties in the mixture, with and without priming.

S1 Lower dimension models

S1.1 Common notations

The total host density is N, a constant. The number of varieties in the mixture is n. The varieties correspond to host genotypes with a single resistance gene, R_i, $i = 1, ..., n$. All varieties are present in the same proportion $p = \frac{1}{n}$.

The density of uninfected hosts of variety R_i is S_i, for $i = 1, ..., n$. Similarly, the density of primed hosts of variety R_i is S_i^*, for $i = 1, ..., n$.

The transmission rate, β, is the same for all pathogen genotypes. The virulence cost, $0 \leq c \leq 1$, is the same for all virulence alleles. The virulence cost is multiplicative, meaning that if a pathogen genotype has k virulence alleles, it bears a fitness cost $(1-c)^k$. We note that $1 \leq k \leq n$ is termed the virulence complexity of the pathogen genotype. The priming efficiency, $0 \leq \rho \leq 1$, reduces the probability that a host is infected by a virulent pathogen genotype. The rate at which priming loses its efficiency is γ. The rate at which a host is replaced with an uninfected one (due to harvesting and replanting) is α.

Model parameters and variables are listed in Table S1.

S1.2 Model for $n = 2$ varieties

The first step in building our model is to consider the simplest host mixture, one with only two resistant varieties. The outcome of the possible host-pathogen interactions are summarized in Table S2. Each resistant variety corresponds to a single resistance gene (either R_1 or R_2). There are three possible pathogen genotypes:

Parameter	Definition
n	number of resistant varieties in the mixture
R_i	variety with a single resistance gene at locus $i = 1, \dots, n$
p	proportion of each variety in the mixture: $p = 1/n$
k	virulence complexity of a pathogen genotype: $k = 1, \dots, n$
c	virulence cost: $c \in [0, 1]$
ρ	priming effect: $\rho \in [0, 1]$
γ	priming loss rate: $\gamma \geq 0$
α	harvest and replanting rate: $\alpha > 0$
β	pathogen transmission rate: $\beta > 0$
N	total host population density: $N > 0$
R	basic reproduction number: $R = \beta N/\alpha > 1$
ν	dimensionless parameter: $\nu = (\gamma + \alpha)/\alpha \geq 1$
**Variable**	**Definition**
t	time: $t \geq 0$
I_i	density of hosts of variety i infected by the associated singly virulent pathogen genotype
J_i	density of hosts of variety i infected by a doubly virulent pathogen genotype
G_i	density of hosts of variety i infected by the triply virulent pathogen genotype (for $n = 3$)
S_i^*	density of hosts of variety i that are primed
S_i	density of hosts of variety i that are uninfected
y_i	proportion of hosts of variety i infected by the associated singly virulent pathogen: $y_i = I_i/N$
z_i	proportion of hosts of variety i infected by a doubly virulent pathogen: $z_i = J_i/N$
m_i	proportion of hosts of variety i that are primed: $m = S_i^*/N$
x_k	proportion of hosts of the focal variety infected by a pathogen of virulence complexity k
m	proportion of hosts of the focal variety that are primed
X	proportion of hosts of the focal variety that are neither infected nor primed

Table S1: Model parameters and variables.

$\mathrm{av}_1/\mathrm{Av}_2$, $\mathrm{Av}_1/\mathrm{av}_2$, and $\mathrm{av}_1/\mathrm{av}_2$ (Av means "avirulent" and av means "virulent"). For instance, $\mathrm{Av}_1/\mathrm{av}_2$ means avirulent with respect to R_1 and virulent with respect to R_2. More specifically, $\mathrm{Av}_1/\mathrm{av}_2$ cannot infect R_1, but instead triggers immune priming on R_1. Symmetrically, the other singly virulent pathogen genotype, $\mathrm{av}_1/\mathrm{Av}_2$, triggers immune priming on R_2. In contrast, $\mathrm{av}_1/\mathrm{av}_2$ means that this pathogen genotype is able to infect both R_1 and R_2: this is a doubly virulent pathogen genotype. We ig-

Variety \ Pathogen	av_1/Av_2	Av_1/av_2	av_1/av_2
R_1	1	**priming**	1
R_2	**priming**	1	1

Table S2: The symbol 1 means infection.

nore doubly avirulent (Av_1/Av_2) pathogen genotypes since they cannot infect both varieties, and therefore cannot invade the host mixture considered.

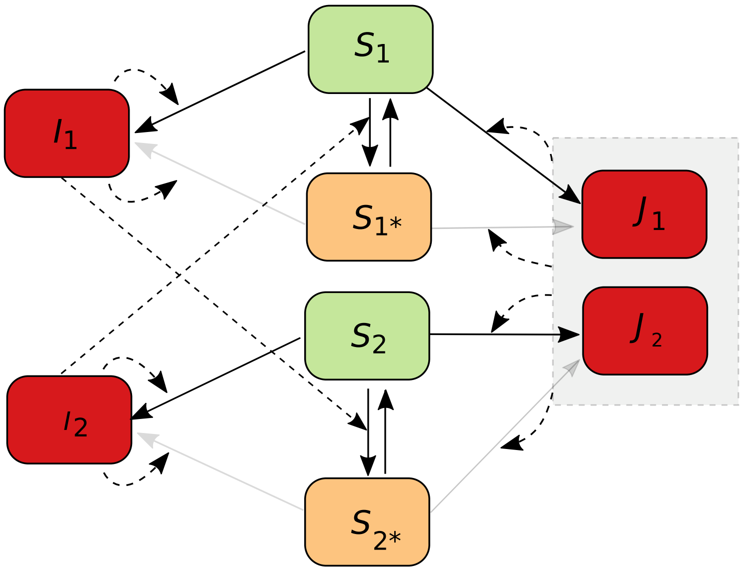

Figure S1: Simplified compartmental diagram for the epidemiological model (S1). The model notations and their definitions are listed in Table S1.

The density of hosts of variety R_i infected by the corresponding singly virulent pathogen genotype is I_i, for $i = 1, 2$. The density of hosts of variety R_i infected by the doubly virulent pathogen genotype is J_i, for $i = 1, 2$. The density of uninfected hosts of variety R_i is therefore $S_i = pN - S_i^* - I_i - J_i$, for $i = 1, 2$.

The model depicted in Figure S1 is formulated as a system of ordinary differential

equations, in which the dot denotes differentiation with respect to time t:

$$
\begin{aligned}
\dot{I}_1 &= (1-c)\beta I_1 S_1 + (1-\rho)(1-c)\beta I_1 S_1^* - \alpha I_1\,, \\
\dot{I}_2 &= (1-c)\beta I_2 S_2 + (1-\rho)(1-c)\beta I_2 S_2^* - \alpha I_2\,, \\
\dot{J}_1 &= (1-c)^2\beta (J_1+J_2) S_1 + (1-\rho)(1-c)^2\beta (J_1+J_2) S_1^* - \alpha J_1\,, \\
\dot{J}_2 &= (1-c)^2\beta (J_1+J_2) S_2 + (1-\rho)(1-c)^2\beta (J_1+J_2) S_2^* - \alpha J_2\,, \\
\dot{S}_1^* &= (1-c)\beta I_2 S_1 - (1-\rho)\beta S_1^* \left((1-c)I_1 + (1-c)^2(J_1+J_2)\right) - (\gamma+\alpha)S_1^*\,, \\
\dot{S}_2^* &= (1-c)\beta I_1 S_2 - (1-\rho)\beta S_2^* \left((1-c)I_2 + (1-c)^2(J_1+J_2)\right) - (\gamma+\alpha)S_2^*\,.
\end{aligned}
\tag{S1}
$$

We re-scale variables and parameters in this way:

$$
\begin{aligned}
y_1 &= \frac{I_1}{N}\,, \quad y_2 = \frac{I_2}{N}\,, \quad z_1 = \frac{J_1}{N}\,, \quad z_2 = \frac{J_2}{N}\,, \quad m_1 = \frac{S_1^*}{N}\,, \\
m_2 &= \frac{S_2^*}{N}\,, \quad t^* = \alpha t\,, \quad R = \frac{\beta N}{\alpha}\,, \quad \nu = \frac{(\gamma+\alpha)}{\alpha}\,.
\end{aligned}
$$

In dimensionless form, uninfected hosts densities are $s_1 = \frac{1}{2} - m_1 - y_1 - z_1$ and $s_2 = \frac{1}{2} - m_2 - y_2 - z_2$. A dimensionless version of model (S1) is the following, where the prime denotes differentiation with respect to t^*:

$$
\begin{aligned}
y_1' &= (1-c)R y_1 s_1 + (1-\rho)(1-c)R y_1 m_1 - y_1\,, \\
y_2' &= (1-c)R y_2 s_2 + (1-\rho)(1-c)R y_2 m_2 - y_2\,, \\
z_1' &= (1-c)^2 R (z_1+z_2) s_1 + (1-\rho)(1-c)^2 R (z_1+z_2) m_1 - z_1\,, \\
z_2' &= (1-c)^2 R (z_1+z_2) s_2 + (1-\rho)(1-c)^2 R (z_1+z_2) m_2 - z_2\,, \\
m_1' &= (1-c)R y_2 s_1 - (1-\rho)R m_1 \left((1-c)y_1 + (1-c)^2(z_1+z_2)\right) - \nu m_1\,, \\
m_2' &= (1-c)R y_1 s_2 - (1-\rho)R m_2 \left((1-c)y_2 + (1-c)^2(z_1+z_2)\right) - \nu m_2\,.
\end{aligned}
\tag{S2}
$$

Figure S2A shows that regardless of the initial conditions on each variety, the infection dynamics of both varieties may converge and become identical (meaning that $y_1 - y_2 \to 0$, $z_1 - z_2 \to 0$, and $m_1 - m_2 \to 0$). Thus, the above model (S2) may be reduced such that the epidemic dynamics are entirely described by keeping track of a single variety.

Next, we assume that both varieties share the same initial conditions, that is $y_i(0) = y_0$, $z_i(0) = z_0$, and $m_i(0) = m_0$, for $i = 1, 2$. In this way, their dynamics are

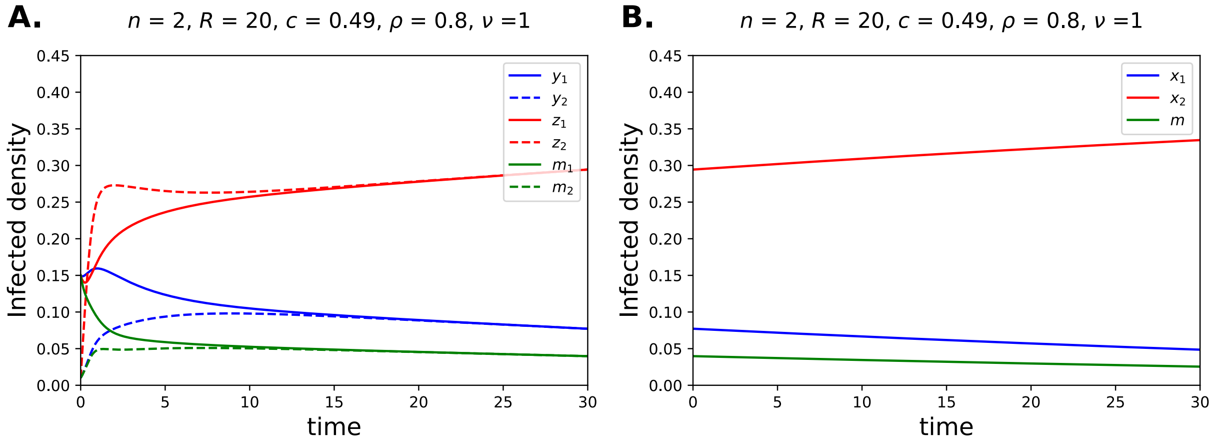

Figure S2: Infection dynamics over time for a model with $n = 2$ varieties. **A** shows a simulation from the full model (S2). The full lines correspond to variety 1, and the dashed lines correspond to variety 2. The density of hosts of variety i infected by the corresponding singly virulent pathogen genotype is y_i, $i = 1, 2$. The density of hosts of variety i infected by the doubly virulent pathogen genotype is z_i, $i = 1, 2$. The density of hosts of variety i that are primed is m_i, $i = 1, 2$. The initial conditions were arbitrarily chosen as $y_1 = z_1 = m_1 = 0.15$, and $y_2 = z_2 = m_2 = 0.01$. **B** shows a simulation from the reduced model (S3), i.e. the model keeping track of a single focal variety. The density of hosts of the focal variety infected by the corresponding singly virulent pathogen genotype is x_1. The density of hosts of the focal variety infected by the doubly virulent pathogen genotype is x_2. The density of hosts of the focal variety that are primed is m. The initial conditions in **B** are the final conditions (at time $t = 30$) in **A**.

identical, meaning that $y_1(t) = y_2(t)$, $z_1(t) = z_2(t)$, and $m_1(t) = m_2(t)$ for all t.

Let us focus on one of both varieties, hereafter the focal variety. In what follows, we denote by x_k the density of hosts of the focal variety that are infected by one pathogen genotype of virulence complexity $k = 1, 2$. The density of hosts of the focal variety infected by the corresponding singly virulent pathogen genotype is x_1. The density of hosts of the focal variety infected by the doubly virulent pathogen genotype is x_2. The density of hosts of the focal variety that are primed is m.

The reduced model is:

$$
\begin{aligned}
x_1' &= x_1\left(R(1-c)\left(\left(\frac{1}{2}-m-x_1-x_2\right)+(1-\rho)m\right)-1\right), \\
x_2' &= x_2\left(2R(1-c)^2\left(\left(\frac{1}{2}-m-x_1-x_2\right)+(1-\rho)m\right)-1\right), \\
m' &= \left(\frac{1}{n}-m-x_1-x_2\right)R(1-c)x_1-(1-\rho)m\left(R(1-c)x_1+R(1-c)^2 2x_2\right)-\nu m.
\end{aligned}
\tag{S3}
$$

Figure S2B shows that the reduced model (S3) indeed extrapolates the dynamics from the full model (S2).

Variety \ Pathogen	$av_1/Av_2/Av_3$	$Av_1/av_2/Av_3$	$Av_1/Av_2/av_3$	$av_1/av_2/Av_3$	$av_1/Av_2/av_3$	$Av_1/av_2/av_3$	$av_1/av_2/av_3$
R_1	1	**priming**	**priming**	1	1	**priming**	1
R_2	**priming**	1	**priming**	1	**priming**	1	1
R_3	**priming**	**priming**	1	**priming**	1	1	1

Table S3: The symbol 1 means infection.

S1.3 Model for $n = 3$ varieties

The model is extended to $n = 3$ varieties. The outcome of the possible host-pathogen interactions are summarized in Table S3. Each resistant variety corresponds to a single resistance gene (either R_1, R_2 or R_3). There are seven possible pathogen genotypes: $av_1/Av_2/Av_3$, $Av_1/av_2/Av_3$, $Av_1/Av_2/av_3$, $av_1/av_2/Av_3$, $av_1/Av_2/av_3$, $Av_1/av_2/av_3$ and $av_1/av_2/av_3$ (Av means “avirulent” and av means “virulent”). For instance, $av_1/av_2/av_3$ means that this pathogen genotype is able to infect R_1, and R_2, and R_3: this is a triply virulent pathogen genotype. In contrast, $av_1/av_2/Av_3$, $av_1/Av_2/av_3$, $Av_1/av_2/av_3$ cannot infect R_3, R_2 and R_1, respectively, but instead trigger immune priming on R_3, R_2, and R_1, respectively. They are doubly virulent. By definition, singly virulent pathogen genotypes $av_1/Av_2/Av_3$, $Av_1/av_2/Av_3$, $Av_1/Av_2/av_3$ are able to infect R_1, R_2 and R_3, and trigger priming on R_2 and R_3, R_1 and R_3, and R_2 and R_3, respectively. We ignore triply avirulent ($Av_1/Av_2/Av_3$) pathogen genotypes since they cannot infect any variety, and therefore cannot invade this host mixture.

The density of hosts of variety R_i infected by the corresponding singly virulent pathogen genotype is I_i, for $i = 1,2,3$. The density of hosts of variety R_i infected by a doubly virulent pathogen genotype having virulence alleles i and j is J_{ij}, for $i,j = 1,2,3$ and $j \neq i$. For example, J_{12} corresponds to hosts of variety R_1 infected by the pathogen genotype $\alpha v_1/\alpha v_2/v_3$, whereas J_{21} corresponds to hosts of variety R_2 infected by the same pathogen genotype. The density of hosts of variety R_i infected by the triply virulent pathogen genotype is G_i, for $i = 1,2,3$. The density of uninfected hosts of variety R_i is therefore $S_i = pN - S_i^* - I_i - J_{ij} - J_{ik} - G_i$, for $i = 1,2,3$, $i \neq j \neq k$.

The model is formulated as a system of ordinary differential equations, in which the dot denotes differentiation with respect to time t:

$$
\begin{aligned}
\dot{I}_1 &= (1-c)\beta I_1 S_1 + (1-\rho)(1-c)\beta I_1 S_1^* - \alpha I_1\,, \\
\dot{I}_2 &= (1-c)\beta I_2 S_2 + (1-\rho)(1-c)\beta I_2 S_2^* - \alpha I_2\,, \\
\dot{I}_3 &= (1-c)\beta I_3 S_3 + (1-\rho)(1-c)\beta I_3 S_3^* - \alpha I_3\,, \\
\dot{J}_{1,2} &= (1-c)^2\beta(J_{1,2}+J_{2,1})S_1 + (1-\rho)(1-c)^2\beta(J_{1,2}+J_{2,1})S_1^* - \alpha J_{1,2}\,, \\
\dot{J}_{1,3} &= (1-c)^2\beta(J_{1,3}+J_{3,1})S_1 + (1-\rho)(1-c)^2\beta(J_{1,3}+J_{3,1})S_1^* - \alpha J_{1,3}\,, \\
\dot{J}_{2,1} &= (1-c)^2\beta(J_{1,2}+J_{2,1})S_2 + (1-\rho)(1-c)^2\beta(J_{1,2}+J_{2,1})S_2^* - \alpha J_{2,1}\,, \\
\dot{J}_{2,3} &= (1-c)^2\beta(J_{2,3}+J_{3,2})S_2 + (1-\rho)(1-c)^2\beta(J_{2,3}+J_{3,2})S_2^* - \alpha J_{2,3}\,, \\
\dot{J}_{3,1} &= (1-c)^2\beta(J_{3,1}+J_{1,3})S_3 + (1-\rho)(1-c)^2\beta(J_{3,1}+J_{1,3})S_3^* - \alpha J_{3,1}\,, \\
\dot{J}_{3,2} &= (1-c)^2\beta(J_{3,2}+J_{2,3})S_3 + (1-\rho)(1-c)^2\beta(J_{3,2}+J_{2,3})S_3^* - \alpha J_{3,2}\,, \qquad \text{(S4)} \\
\dot{G}_1 &= (1-c)^3\beta(G_1+G_2+G_3)S_1 + (1-\rho)(1-c)^3\beta(G_1+G_2+G_3)S_1^* - \alpha G_1\,, \\
\dot{G}_2 &= (1-c)^3\beta(G_1+G_2+G_3)S_2 + (1-\rho)(1-c)^3\beta(G_1+G_2+G_3)S_2^* - \alpha G_2\,, \\
\dot{G}_3 &= (1-c)^3\beta(G_1+G_2+G_3)S_3 + (1-\rho)(1-c)^3\beta(G_1+G_2+G_3)S_3^* - \alpha G_3\,, \\
\dot{S}_1^* &= (1-c)\beta(I_2+I_3)S_1 + (1-c)^2\beta(J_{2,3}+J_{3,2})S_1 - (1-\rho)(1-c)\beta I_1 S_1^* \\
&\quad -(1-\rho)(1-c)^2\beta(J_{1,2}+J_{2,1})S_1^* - (1-\rho)(1-c)^2\beta(J_{1,3}+J_{3,1})S_1^* \\
&\quad -(1-\rho)(1-c)^3\beta(G_1+G_2+G_3)S_1^* - (\gamma+\alpha)S_1^*\,, \\
\dot{S}_2^* &= (1-c)\beta(I_1+I_3)S_2 + (1-c)^2\beta(J_{1,3}+J_{3,1})S_2 - (1-\rho)(1-c)\beta I_2 S_2^* \\
&\quad -(1-\rho)(1-c)^2\beta(J_{1,2}+J_{2,1})S_2^* - (1-\rho)(1-c)^2\beta(J_{2,3}+J_{3,2})S_2^* \\
&\quad -(1-\rho)(1-c)^3\beta(G_1+G_2+G_3)S_2^* - (\gamma+\alpha)S_2^*\,, \\
\dot{S}_3^* &= (1-c)\beta(I_1+I_2)S_3 + (1-c)^2\beta(J_{1,2}+J_{2,1})S_3 - (1-\rho)(1-c)\beta I_3 S_3^* \\
&\quad -(1-\rho)(1-c)^2\beta(J_{1,3}+J_{3,1})S_3^* - (1-\rho)(1-c)^2\beta(J_{2,3}+J_{3,2})S_3^* \\
&\quad -(1-\rho)(1-c)^3\beta(G_1+G_2+G_3)S_3^* - (\gamma+\alpha)S_3^*\,.
\end{aligned}
$$

We re-scale variables and parameters in this way:

$$
\begin{aligned}
y_1 &= \frac{I_1}{N}\,, \quad y_2 = \frac{I_2}{N}\,, \quad y_3 = \frac{I_3}{N}\,, \quad z_{1,2} = \frac{J_{1,2}}{N}\,, \quad z_{1,3} = \frac{J_{1,3}}{N}\,, \quad z_{2,1} = \frac{J_{2,1}}{N}\,, \\
z_{2,3} &= \frac{J_{2,3}}{N}\,, \quad z_{3,1} = \frac{J_{3,1}}{N}\,, \quad z_{3,2} = \frac{J_{3,2}}{N}\,, \quad w_1 = \frac{G_1}{N}\,, \quad w_2 = \frac{G_2}{N}\,, \quad w_3 = \frac{G_3}{N}\,, \\
m_1 &= \frac{S_1^*}{N}\,, \quad m_2 = \frac{S_2^*}{N}\,, \quad m_3 = \frac{S_3^*}{N}\,, \quad t^* = \alpha t\,, \quad R = \frac{\beta N}{\alpha}\,, \quad \nu = \frac{(\gamma+\alpha)}{\alpha}\,.
\end{aligned}
$$

In dimensionless form, uninfected hosts densities are $s_1 = \frac{1}{3} - m_1 - y_1 - z_{1,2} - z_{1,3} - w_1$, $s_2 = \frac{1}{3} - m_2 - y_2 - z_{2,1} - z_{2,3} - w_2$, and $s_3 = \frac{1}{3} - m_3 - y_3 - z_{3,1} - z_{3,2} - w_3$. A dimensionless version of model (S4) is the following, where the prime denotes differentiation with respect to t^*:

$$
\begin{aligned}
y_1' &= (1-c)Ry_1\left(\frac{1}{3} - m_1 - y_1 - z_{1,2} - z_{1,3} - w_1\right) + (1-\rho)(1-c)Ry_1m_1 - y_1\,, \\
y_2' &= (1-c)Ry_2\left(\frac{1}{3} - m_2 - y_2 - z_{2,1} - z_{2,3} - w_2\right) + (1-\rho)(1-c)Ry_2m_2 - y_2\,, \\
y_3' &= (1-c)Ry_3\left(\frac{1}{3} - m_3 - y3 - z_{3,1} - z_{3,2} - w_3\right) + (1-\rho)(1-c)Ry_3m_3 - y_3\,, \\
z_{1,2}' &= (1-c)^2R(z_{1,2}+z_{2,1})\left(\frac{1}{3} - y_1 - y_1 - z_{1,2} - z_{1,3} - w_1\right) + (1-\rho)(1-c)^2R(z_{1,2}+z_{2,1})m_1 - z_{1,2}\,, \\
z_{1,3}' &= (1-c)^2R(z_{1,3}+z_{3,1})\left(\frac{1}{3} - m_1 - y_1 - z_{1,2} - z_{1,3} - w_1\right) + (1-\rho)(1-c)^2R(z_{1,3}+z_{3,1})m_1 - z_{1,3}\,, \\
z_{2,1}' &= (1-c)^2R(z_{1,2}+z_{2,1})\left(\frac{1}{3} - m_2 - y_2 - z_{2,1} - z_{2,3} - w_2\right) + (1-\rho)(1-c)^2R(z_{1,2}+z_{2,1})m_2 - z_{2,1}\,, \\
z_{2,3}' &= (1-c)^2R(z_{2,3}+z_{3,2})\left(\frac{1}{3} - m_2 - y_2 - z_{2,1} - z_{2,3} - w_2\right) + (1-\rho)(1-c)^2R(z_{2,3}+z_{3,2})m_2 - z_{2,3}\,, \\
z_{3,1}' &= (1-c)^2R(z_{1,3}+z_{3,1})\left(\frac{1}{3} - m_3 - y_3 - z_{3,1} - z_{1,3} - w_3\right) + (1-\rho)(1-c)^2R(z_{1,3}+z_{3,1})m_3 - z_{3,1}\,, \\
z_{3,2}' &= (1-c)^2R(z_{1,3}+z_{3,1})\left(\frac{1}{3} - m_3 - y_3 - z_{3,2} - z_{3,2} - w_3\right) + (1-\rho)(1-c)^2R(z_{2,3}+z_{3,2})m_3 - z_{3,2}\,, \qquad \text{(S5)} \\
w_1' &= (1-c)^3R(w_1+w_2+w_3)\left(\frac{1}{3} - m_1 - y_1 - z_{1,2} - z_{1,3} - w_1\right) + (1-\rho)(1-c)^3Rw_1m_1 - w_1\,, \\
w_2' &= (1-c)^3R(w_1+w_2+w_3)\left(\frac{1}{3} - m_2 - y_2 - z_{2,1} - z_{2,3} - w_2\right) + (1-\rho)(1-c)^3Rw_2m_2 - w_2\,, \\
w_3' &= (1-c)^3R(w_1+w_2+w_3)\left(\frac{1}{3} - m_3 - y_3 - z_{1,3} - z_{3,2} - w_3\right) + (1-\rho)(1-c)^3Rw_3m_3 - w_3\,, \\
m_1' &= (1-c)R(y_2+y_3)\left(\frac{1}{3} - m_1 - y_1 - z_{1,2} - z_{1,3} - w_1\right) \\
&\quad +(1-c)^2R(z_{2,3}+z_{3,2})\left(\frac{1}{3} - m_1 - y_1 - z_{1,2} - z_{1,3} - w_1\right) \\
&\quad -(1-\rho)(1-c)Ry_1m_1 - (1-\rho)(1-c)^2R(z_{1,2}+z_{2,1})m_1 \\
&\quad -(1-\rho)(1-c)^2R(z_{1,3}+z_{3,1})m_1 - (1-\rho)(1-c)^3R(w_1+w_2+w_3)m_1 - \nu m_1\,, \\
m_2' &= (1-c)R(y_1+y_3)\left(\frac{1}{3} - m_2 - y_2 - z_{2,1} - z_{2,3} - w_2\right) \\
&\quad +(1-c)^2R(z_{1,3}+z_{3,1})\left(\frac{1}{3} - m_2 - y_2 - z_{2,1} - z_{2,3} - w_2\right) \\
&\quad -(1-\rho)(1-c)Ry_2m_2 - (1-\rho)(1-c)^2R(z_{1,2}+z_{2,1})m_2 \\
&\quad -(1-\rho)(1-c)^2R(z_{2,3}+z_{3,2})m_2 - (1-\rho)(1-c)^3R(w_1+w_2+w_3)m_2 - \nu m_2\,, \\
m_3' &= (1-c)R(y_1+y_2)\left(\frac{1}{3} - m_3 - y_3 - z_{3,1} - z_{3,2} - w_3\right) \\
&\quad +(1-c)^2R(z_{1,2}+z_{2,1})\left(\frac{1}{3} - m_3 - y_3 - z_{3,1} - z_{3,2} - w_3\right) \\
&\quad -(1-\rho)(1-c)Ry_3m_3 - (1-\rho)(1-c)^2R(z_{1,3}+z_{3,1})m_3 \\
&\quad -(1-\rho)(1-c)^2R(z_{2,3}+z_{3,2})m_3 - (1-\rho)(1-c)^3R(w_1+w_2+w_3)m_3 - \nu m_3\,.
\end{aligned}
$$

Figure S3 shows that regardless of the initial conditions on each variety, the infection dynamics of all varieties may converge and become identical (meaning

$y_i - y_j \to 0$, $z_{ij} - z_{kl} \to 0$, $w_i - w_j \to 0$, and $m_i - m_j \to 0$), for all $i, j, k, l = 1, 2, 3$ such that $i \neq j$ and $k \neq l$. Thus, the above model (S5) may be reduced such that the epidemic dynamics are entirely described by keeping track of a single variety.

Next, we assume that all varieties share the same initial conditions, that is $y_i(0) = y_0$, $z_{ij}(0) = z_0$, $w_i(0) = w_0$ and $m_i(0) = m_0$, for all $i, j = 1, 2, 3$ and $j \neq i$. In that way, their dynamics are identical, meaning that $y_i(t) = y_j(t)$, $z_{ij}(t) = z_{kl}(t)$, $w_i(t) = w_j(t)$, and $m_i(t) = m_j(t)$ for all t, and for all $i, j, k, l = 1, 2, 3$ such that $i \neq j$ and $k \neq l$.

Let us focus on one of the three varieties, hereafter the focal variety. In what follows, we denote by x_k the density of hosts of the focal variety that are infected by one pathogen genotype of virulence complexity $k = 1, 2, 3$. The density of hosts of the focal variety infected by the corresponding singly virulent pathogen genotype is x_1. The density of hosts of the focal variety infected by one of the corresponding doubly virulent pathogen genotypes is x_2. The density of hosts of the focal variety infected by the triply virulent pathogen genotype is x_3. The density of hosts of the focal variety that are primed is m.

The reduced model is:

$$\begin{aligned}
x_1' &= R(1-c)x_1\left(\frac{1}{3} - m - x_1 - 2x_2 - x_3\right) + (1-\rho)R(1-c)x_1 m - x_1\,,\\
x_2' &= R(1-c)^2 2x_2\left(\frac{1}{3} - m - x_1 - 2x_2 - x_3\right) + (1-\rho)R(1-c)^2 2x_2 m - x_2\,,\\
x_3' &= R(1-c)^3 3x_3\left(\frac{1}{3} - m - x_1 - 2x_2 - x_3\right) + (1-\rho)R(1-c)^3 3x_3 m - x_3\,,\\
m' &= 2R\left(\frac{1}{3} - m - x_1 - 2x_2 - x_3\right)\big((1-c)x_1 + (1-c)^2 x_2\big)\\
&\quad -(1-\rho)Rm\big((1-c)x_1 + (1-c)^2 4x_2 + (1-c)^3 3x_3\big) - \nu m\,.
\end{aligned} \tag{S6}$$

Figure S3 shows that the reduced model (S6) indeed extrapolates the dynamics from the full model (S5).

S2 Model for an arbitrary number n of varieties

The model is extended to n varieties based on the reduced models (S3) and (S6). The number of varieties in the mixture is n. The virulence complexity is $k = 1, \ldots, n$.

We focus on an arbitrary variety, hereafter the focal variety. The density of hosts

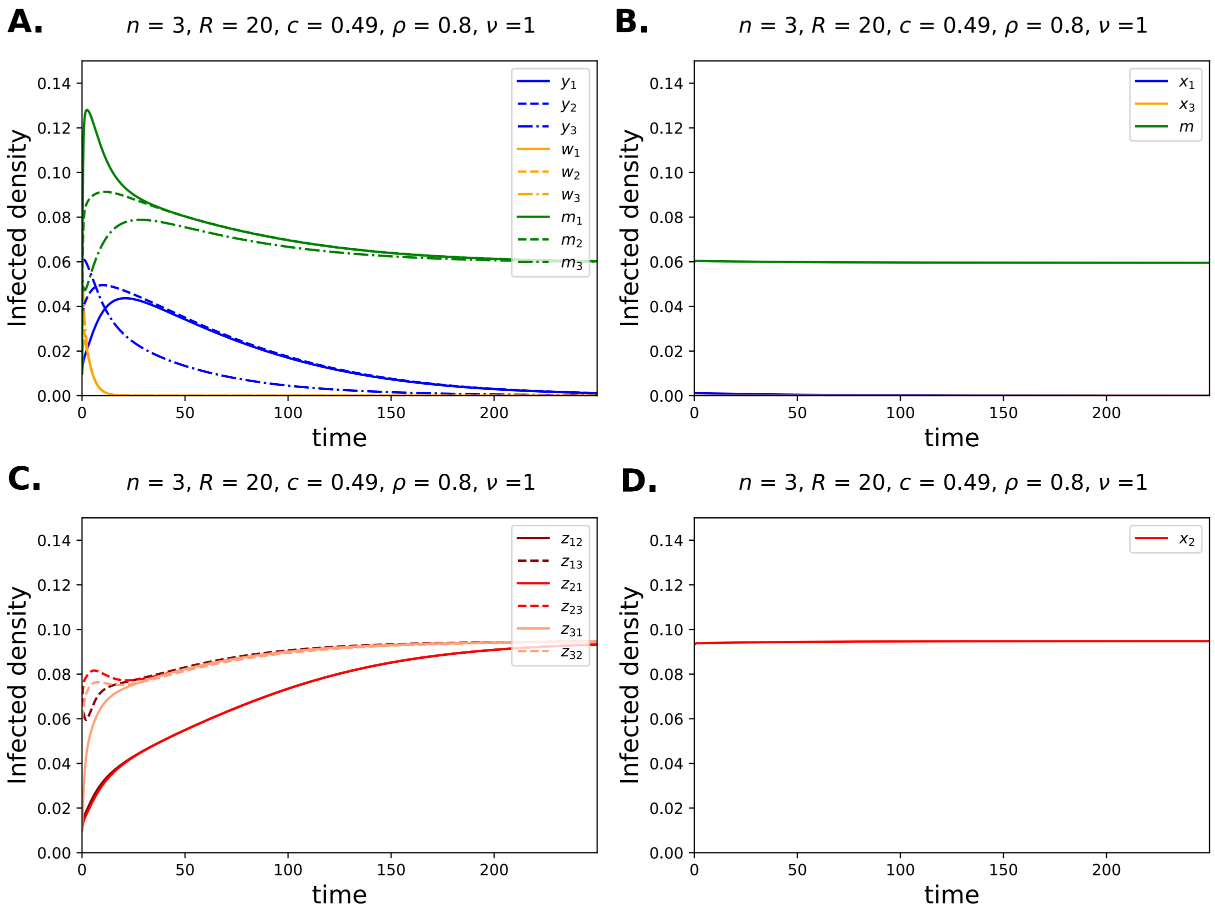

Figure S3: Infection dynamics over time for a model with $n = 3$ varieties. For clarity, the infection dynamics of hosts infected with pathogen genotypes having 2 virulence alleles have been separated on graphs **C** and **D**. **A** and **C** show the full model (S4). The full lines correspond to variety R_1, the dashed lines to variety R_2, and the dot-dashed lines to variety R_3. The density of hosts of variety R_i infected by a singly virulent pathogen genotype is y_i, $i = 1, 2, 3$. The density of hosts of variety R_i infected by a doubly pathogen genotype is z_{ij}, $i, j = 1, 2, 3$ and $i \neq j$. The density of hosts of variety R_i infected by the triply pathogen genotype is w_i, $i = 1, 2, 3$. The density of hosts of variety R_i that are primed is m_i, $i = 1, 2, 3$. Initial conditions are arbitrary. **B** and **D** show the reduced model (S6), i.e. the model for a focal variety. The density of hosts of the focal variety infected by the corresponding singly virulent pathogen genotype is x_1. The density of hosts of the focal variety infected by one of the corresponding doubly virulent genotypes is x_2. The density of hosts of the focal variety infected by the triply virulent pathogen genotype is x_3. Initial conditions in **B** and **D** are the final conditions (at time $t = 250$) in **A** and **C**, respectively.

of the focal variety infected by a single pathogen genotype with a given virulence complexity k is x_k. A pathogen genotype of complexity k is able to infect k different varieties because for each virulence allele, it infects a different variety; therefore its density is kx_k.

The infection force of a given pathogen genotype having virulence complexity k is f_k. Its per capita transmission rate is $R(1-c)^k$ and its density is kx_k. Therefore,

$$f_k = kR(1-c)^k x_k \,. \tag{S7}$$

To infect the focal variety, a pathogen of complexity k must carry the virulence allele specific to that host, as well as $k-1$ other virulence alleles among the $n-1$ remaining loci. Therefore, there are $\binom{n-1}{k-1}$ different pathogen genotypes of virulence complexity k able to infect the focal variety.

The density of uninfected and unprimed hosts for the focal variety is defined as X:

$$X = \frac{1}{n} - m - \underbrace{\sum_{i=1}^{n} \binom{n-1}{i-1} x_i}_{A} . \tag{S8}$$

The term $\frac{1}{n}$ represents the total density of hosts of the focal variety. The density of primed hosts for the focal variety is m. The term A corresponds to the total density of infected hosts among the focal variety. This is the sum, over all possible virulence complexities, of the number of pathogen genotypes of complexity i able to infect the focal variety, $\binom{n-1}{i-1}$, multiplied by their individual density on the focal variety x_i.

The infection force of the pathogen population on the focal variety is F. This is the density of pathogen genotypes that can infect the focal variety. This is the sum, over all possible virulence complexities, of the number of pathogen genotypes of complexity i able to infect the focal variety, $\binom{n-1}{i-1}$, multiplied by their individual infection force on the focal variety, f_i:

$$F = \sum_{i=1}^{n} \binom{n-1}{i-1} f_i . \tag{S9}$$

The priming force is P. This is the density of pathogen genotypes that can trigger priming on the focal variety. To prime the focal variety, a pathogen of complexity k must not carry the virulence allele specific to that host, and carry k alleles among $n-1$ loci to be of complexity k. Therefore, there are $\binom{n-1}{k}$ different pathogen genotypes of virulence complexity k able to prime the focal variety. The most complex pathogen genotype x_n can infect all varieties and therefore never triggers priming. Therefore, P is the sum, over the remaining $n-1$ virulence complexities, of the number of pathogen genotypes of complexity i and able to prime the focal variety, $\binom{n-1}{i}$, times their individual infection force on the focal variety, f_i:

$$P = \sum_{i=1}^{n-1} \binom{n-1}{i} f_i . \tag{S10}$$

The model is composed of n x_k-equations, since $k = 1, \ldots, n$, plus 1 equation for primed hosts m. The $n+1$ dimensional model is therefore:

$$\begin{aligned} x_k' &= f_k\,(X + (1-\rho)m) - x_k\,, \\ m' &= XP - (1-\rho)mF - \nu m\,. \end{aligned} \tag{S11}$$

S3 Model analysis

Model (S11) is equivalently expressed as: for $k = 1, .., n$,

$$\begin{aligned} x_k' &= x_k\,(\phi_k\,(X + (1-\rho)m) - 1)\,, \\ m' &= X \sum_{i=1}^{n-1} \binom{n-1}{i} \phi_i x_i - (1-\rho)m \sum_{i=1}^{n} \binom{n-1}{i-1} \phi_i x_i - \nu m\,, \end{aligned} \tag{S12}$$

In the above equation, ϕ_k denote the pathogen fitness:

$$\phi_k = R(1-c)^k k\,.$$

Theorem 1. *If no two virulence complexities result in the same pathogen fitness, there can be at most one complexity that subsists at equilibrium.*

Proof. If $x_k > 0$ for any k in $\{1, \cdots, n\}$, the equilibrium condition $x_k' = 0$ is equivalent to

$$X + (1-\rho)m = \frac{1}{\phi_k}\,. \tag{S13}$$

Proof by contradiction: assume there exists an equilibrium such that at least 2 complexities, $i, j = 1, .., n$, can persist. This implies:

$$X + (1-\rho)m = 1/\phi_i\,, \quad \text{and} \quad X + (1-\rho)m = 1/\phi_j\,.$$

This is impossible unless $\phi_i = \phi_j$, which is a non-generic (biologically irrelevant) case.

□

Therefore, the equilibria are:

- The disease-free equilibrium: for all $k = 1, \ldots, n$, $\bar{x}_k = 0$, and $\bar{m} = 0$.

- $n-1$ potential equilibria such that $(0\ldots,0,\bar{x}_k,0,\ldots,0,\bar{m}_k)$, with $k<n$, $\bar{x}_k>0$, and $\bar{m}_k>0$.

- At most 1 equilibrium such that $(0,\ldots,0,\bar{x}_n,0)$, with $\bar{x}_n>0$.

S3.1 Positiveness conditions

S3.1.1 Positiveness of the $(0,\ldots0,\bar{x}_k,0,\ldots0,\bar{m}_k)$ equilibria

Considering equilibria of the type $(0,\ldots0,\bar{x}_k,0,\ldots0,\bar{m}_k)$ with $k<n$, the model (Eq. S12) simplifies as

$$\begin{aligned} x_k' &= 0 = \bar{x}_k\left(\phi_k(X+(1-\rho)\bar{m}_k)-1\right), \\ m' &= 0 = X\binom{n-1}{k}\phi_k\bar{x}_k-(1-\rho)\bar{m}_k\binom{n-1}{k-1}\phi_k\bar{x}_k-\nu\bar{m}_k, \end{aligned} \tag{S14}$$

where

$$X+(1-\rho)\bar{m}_k=\frac{1}{n}-\binom{n-1}{k-1}\bar{x}_k-\rho\bar{m}_k, \tag{S15}$$

after Eq. (S8).

Solving for x at equilibrium. Solving the equation $x_k'=0$ in Eq. (S14) assuming $\bar{x}_k>0$, we obtain

$$\bar{m}_k=\frac{\phi_k\left(\frac{1}{n}-\binom{n-1}{k-1}\bar{x}_k\right)-1}{\rho\phi_k}, \tag{S16}$$

which, assuming $\bar{x}_k>0$, implies

$$\bar{m}_k<\frac{\frac{\phi_k}{n}-1}{\rho\phi_k}. \tag{S17}$$

A necessary condition for $\bar{x}_k,\bar{m}_k>0$ is therefore:

$$\phi_k>n. \tag{S18}$$

Using the expression of $\bar{m}_k$ given by Eq. (S16) in the m-equation of the simplified model (S14) and rearranging yields, at equilibrium:

$$m'=a_2x_k^2+a_1x+a_0=0, \tag{S19}$$

where the coefficients of the quadratic are defined as:

$$\begin{aligned} a_2 &= \frac{\binom{n-1}{k-1}\phi_k(1-\rho)\left(\binom{n-1}{k}+\binom{n-1}{k-1}\right)}{\rho} > 0\,, \\ a_1 &= \frac{((\nu-\rho+1)n-\phi_k(1-\rho))\binom{n-1}{k-1}+\binom{n-1}{k}(n-\phi_k(1-\rho))}{n\rho}\,, \\ a_0 &= -\frac{\nu(\phi_k-n)}{n\phi_k\rho} < 0\,, \end{aligned}$$

since we previously identified the necessary condition $\phi_k > n$ (Eq. S18).

Since $a_2 > 0$, the quadratic (S19) is U-shaped. And since $a_0 < 0$, it has one positive root and one negative root. The biologically feasible x_k is therefore uniquely defined as the largest root of the quadratic.

Solving for $\bar{m}_k$ at equilibrium. Solving the equation $x'_k = 0$ in Eq. (S14) assuming $\bar{x}_k > 0$, we obtain

$$\bar{x}_k = \frac{\phi_k(1-\bar{m}_k n\rho)-n}{\binom{n-1}{k-1}n\phi_k}\,. \tag{S20}$$

which is equivalent to Eq. (S16).

Using the expression of $\bar{x}_k$ given by Eq. (S20) in the m-equation of the simplified model (S14) and rearranging yields, at equilibrium:

$$m' = b_2\bar{m}_k^2 + b_1\bar{m}_k + b_0 = 0\,, \tag{S21}$$

where the coefficients of the quadratic are defined as:

$$\begin{aligned} b_2 &= \frac{(1-\rho)\binom{n-1}{k}\phi_k\rho}{\binom{n-1}{k-1}} + (1-\rho)\phi_k\rho > 0\,, \\ b_1 &= -\frac{\binom{n-1}{k}((1-\rho)(\phi_k-n)+\rho n)}{\binom{n-1}{k-1}n} - \frac{(1-\rho)(\phi_k-n)}{n} - \nu < 0\,, \\ b_0 &= \frac{\binom{n-1}{k}(\phi_k-n)}{\phi_k\binom{n-1}{k-1}n} > 0\,, \end{aligned}$$

since we previously identified the necessary condition $\phi_k > n$ (Eq. S18).

Since $b_2 > 0$, the quadratic (S21) is U-shaped. Since $b_1 < 0$, its slope at $\bar{m}_k = 0$ is negative, and since $b_0 > 0$, it has either no roots or two positive roots. Since the roots of $\bar{x}_k$ and $\bar{m}_k$ are related (Eq. (S16) shows a linear relationship between $\bar{x}_k$

and $\bar{m}_k$) and since there exists roots for $\bar{x}_k$, then there exists roots for $\bar{m}_k$ that are therefore both positive.

Since there is a single positive root for $\bar{x}_k$, there is a unique pair $(\bar{m}_k, \bar{x}_k)$ where $\bar{m}_k$ and $\bar{x}_k$ are both positive.

After Eq. (S20), we also have

$$\bar{x}_k = \frac{n\phi_k\left(\frac{1}{n} - \bar{m}_k\rho\right) - n}{\binom{n-1}{k-1} n\phi_k} < \frac{n\phi_k\left(\frac{1}{n} - \bar{m}_k\rho\right)}{\binom{n-1}{k-1} n\phi_k} < \frac{1}{n} - \bar{m}_k\rho < \frac{1}{n}\,, \tag{S22}$$

meaning that $\bar{x}_k$ is in the biologically feasible interval $\left(0, \frac{1}{n}\right)$.

Solving for $\bar{m}_k$ in the equation $m' = 0$ in Eq. (S14), we obtain

$$\bar{m}_k = \frac{\binom{n-1}{k}\phi_k\bar{x}_k\left(\frac{1}{n} - \binom{n-1}{k-1}\bar{x}_k\right)}{\phi_k\bar{x}_k\left((1-\rho)\binom{n-1}{k-1} + \binom{n-1}{k}\right) + \nu}\,. \tag{S23}$$

The above equation implies

$$\bar{m}_k < \frac{\binom{n-1}{k}\phi_k\bar{x}_k\left(\frac{1}{n} - \binom{n-1}{k-1}\bar{x}_k\right)}{\phi_k\bar{x}_k\left((1-\rho)\binom{n-1}{k-1} + \binom{n-1}{k}\right)}\,, \tag{S24}$$

which is equivalent to

$$\bar{m}_k < \frac{\binom{n-1}{k}\left(\frac{1}{n} - \binom{n-1}{k-1}\bar{x}_k\right)}{(1-\rho)\binom{n-1}{k-1} + \binom{n-1}{k}} < \frac{\binom{n-1}{k}}{n\left((1-\rho)\binom{n-1}{k-1} + \binom{n-1}{k}\right)} < \frac{\binom{n-1}{k}}{n\binom{n-1}{k}} = \frac{1}{n}\,, \tag{S25}$$

meaning that $\bar{m}_k$ is in the biologically feasible interval $\left(0, \frac{1}{n}\right)$.

S3.1.2 Positiveness of the $(0, \ldots, 0, \bar{x}_n, 0)$ equilibrium

Considering the case in which there is a single nonzero x_k with $k = n$ (and $m = 0$), the model (Eq. S12) simplifies as

$$x_n' = x_n(\phi_n X - 1) = x_n\left(\phi_n\left(\frac{1}{n} - x_n\right) - 1\right)\,. \tag{S26}$$

Therefore, $x_n' = 0$ and $\bar{x}_n > 0$ if and only if

$$\bar{x}_n = \frac{\phi_n - n}{n\phi_n} > 0\,, \tag{S27}$$

which implies $\phi_n > n$.

S3.2 Stability conditions

S3.2.1 Disease-free equilibrium $(0,\dots,0)$

The Jacobian matrix of size $(n+1)\times(n+1)$ evaluated at the Disease-free equilibrium is

$$J = \begin{pmatrix} \frac{\phi_1}{n}-1 & 0 & \dots & \dots & 0 \\ 0 & \ddots & \ddots & \ddots & \vdots \\ \vdots & \ddots & \ddots & \ddots & \vdots \\ 0 & \dots & 0 & \frac{\phi_n}{n}-1 & 0 \\ \frac{\binom{n-1}{1}\phi_1}{n} & \dots & \frac{\binom{n-1}{n-1}\phi_{n-1}}{n} & 0 & -\nu \end{pmatrix}.$$

The Jacobian matrix is a triangular matrix. Therefore, the eigenvalues are its diagonal elements: for all $i = 1,\dots,n$,

$$\begin{aligned} \lambda_i &= \frac{\phi_i}{n} - 1\,, \\ \lambda_{n+1} &= -\nu < 0\,. \end{aligned}$$

Therefore, the Disease-free equilibrium $(0,\dots,0)$ is locally asymptotically stable if and only if $\phi_i < n$ for all $i = 1,\dots,n$.

S3.2.2 The $(0,\dots,0,\bar{x}_k,0,\dots,0,\bar{m}_k)$ equilibrium

We consider the stability of equilibria of the type $(0,\dots,0,\bar{x}_k,0,\dots,0,\bar{m}_k)$ when they are positive, i.e. $\bar{x}_k > 0$ and $\bar{m}_k > 0$. The jacobian matrix of size $(n+1)\times(n+1)$ evaluated around the $(0,\dots,0,\bar{x}_k,0,\dots,0,\bar{m}_k)$ equilibrium is, using Eq. (S13),

$$J=\begin{pmatrix}
\frac{\phi_1}{\phi_k}-1 & 0 & \dots & \dots & \dots & \dots & \dots & \dots & 0\\
0 & \ddots & \ddots & & & & & & \vdots\\
\vdots & \ddots & \ddots & \ddots & & & & & \vdots\\
0 & \dots & 0 & \frac{\phi_{k-1}}{\phi_k}-1 & 0 & \dots & \dots & \dots & 0\\
-\binom{n-1}{0}\phi_k x_k & \dots & \dots & -\binom{n-1}{k-2}\phi_k x_k & -\binom{n-1}{k-1}\phi_k x_k & -\binom{n-1}{k}\phi_k x_k & \dots & -\binom{n-1}{n-1}\phi_k x_k & -\phi_k x_k \rho\\
0 & \dots & \dots & \dots & 0 & \frac{\phi_{k+1}}{\phi_k}-1 & 0 & \dots & 0\\
\vdots & & & & & \ddots & \ddots & \ddots & \vdots\\
0 & \dots & \dots & \dots & \dots & \dots & 0 & \frac{\phi_n}{\phi_k}-1 & 0\\
* & * & * & * & J_{n+1,k} & * & * & * & J_{n+1,n+1}
\end{pmatrix},$$

By switching the k line with the n line of the matrix and the k column with the n column of the matrix, we have a block triangular matrix:

$$\tilde{J}=\left(\begin{array}{cccccccc|cc}
\frac{\phi_1}{\phi_k}-1 & 0 & \dots & \dots & \dots & \dots & \dots & \dots & \dots & 0\\
0 & \ddots & \ddots & & & & & & & \vdots\\
\vdots & \ddots & \ddots & \ddots & & & & & & \vdots\\
\vdots & & \ddots & \frac{\phi_{k-1}}{\phi_k}-1 & \ddots & & & & & \vdots\\
\vdots & & & \ddots & \frac{\phi_n}{\phi_k}-1 & \ddots & & & & \vdots\\
\vdots & & & & \ddots & \frac{\phi_{k+1}}{\phi_k}-1 & \ddots & & & \vdots\\
\vdots & & & & & \ddots & \ddots & \ddots & & \vdots\\
0 & \dots & \dots & \dots & \dots & \dots & 0 & \frac{\phi_{n-1}}{\phi_k}-1 & 0 & 0\\
\hline
-\binom{n-1}{0}\phi_k x_k & \dots & \dots & -\binom{n-1}{k-2}\phi_k x_k & -\binom{n-1}{n-1}\phi_k x_k & -\binom{n-1}{k}\phi_k x_k & \dots & \dots & -\binom{n-1}{k-1}\phi_k x_k & -\phi_k x_k \rho\\
* & * & * & * & * & * & * & * & J_{n+1,k} & J_{n+1,n+1}
\end{array}\right),$$

The Jacobian matrix $\tilde{J}$ has the following form

$$\tilde{J}=\begin{pmatrix} D_1 & 0\\ * & B \end{pmatrix},$$

where D_1 is a $(n-1)\times(n-1)$ diagonal matrix, $*$ is a $2\times(n-1)$ matrix, and B is a 2×2 sub-matrix.

The stability conditions of the $(0,\dots,0,\bar{x}_k,0,\dots,0,\bar{m}_k)$ equilibrium are defined using the determinant and the trace of the sub-matrix B, and the eigenvalues of the matrix D (i.e. its diagonal elements).

The determinant of the matrix D is, after rearranging,

$$\begin{aligned}\det(B) &= \phi_k^2 x_k\left(\binom{n-1}{k-1}\binom{n-1}{k}x_k+\binom{n-1}{k-1}\binom{n-1}{k-1}(1-\rho)x_k+\frac{\binom{n-1}{k-1}\nu}{\phi_k}\right)\\ &+ \phi_k^2 x_k\left(\rho\left(-\binom{n-1}{k-1}\binom{n-1}{k}x_k+X\binom{n-1}{k}-(1-\rho)m\binom{n-1}{k-1}\right)\right). \end{aligned} \tag{S28}$$

Based on $m'=0$ in the m-equation of the simplified model (Eq. S14), we have

$$X\binom{n-1}{k}\phi_k x_k-(1-\rho)m\binom{n-1}{k-1}\phi_k x_k=\nu m\,, \tag{S29}$$

which implies

$$\phi_k x_k\left(X\binom{n-1}{k}-(1-\rho)m\binom{n-1}{k-1}\right)>0\,, \tag{S30}$$

which, using $\phi_k x_k>0$, is equivalent to

$$X\binom{n-1}{k}-(1-\rho)m\binom{n-1}{k-1}>0\,, \tag{S31}$$

which, using the expression of X from Eq. (S15), is equivalent to

$$\left(\frac{1}{n}-m-\binom{n-1}{k-1}x_k\right)\binom{n-1}{k}-(1-\rho)m\binom{n-1}{k-1}>0\,, \tag{S32}$$

which, adding $-\binom{n-1}{k-1}\binom{n-1}{k}x_k$ on both sides of the inequality, is equivalent to

$$-\binom{n-1}{k-1}\binom{n-1}{k}x_k+\left(\frac{1}{n}-m-\binom{n-1}{k-1}x_k\right)\binom{n-1}{k}-(1-\rho)m\binom{n-1}{k-1}>-\binom{n-1}{k-1}\binom{n-1}{k}x_k\,. \tag{S33}$$

We notice that the left-hand-side of the inequality is, using the expression of X from Eq. (S15), exactly the term in factor of ρ in Eq. (S28). Therefore, using (S33) into (S28), we obtain the following inequality:

$$\det(B)>\phi_k^2 x_k\left(\binom{n-1}{k-1}\binom{n-1}{k}x_k+\binom{n-1}{k-1}\binom{n-1}{k-1}(1-\rho)x_k+\frac{\binom{n-1}{k-1}\nu}{\phi_k}-\rho\binom{n-1}{k-1}\binom{n-1}{k}x_k\right), \tag{S34}$$

which, factoring out $\binom{n-1}{k-1}$, is equivalent to

$$\det(B)>\binom{n-1}{k-1}\phi_k^2 x_k\left(\binom{n-1}{k}x_k+\binom{n-1}{k-1}(1-\rho)x_k+\frac{\nu}{\phi_k}-\rho\binom{n-1}{k}x_k\right), \tag{S35}$$

which is equivalent to

$$\det(B)>\binom{n-1}{k-1}\phi_k^2 x_k\left(\binom{n-1}{k}x_k(1-\rho)+\binom{n-1}{k-1}(1-\rho)x_k+\frac{\nu}{\phi_k}\right). \tag{S36}$$

Hence, $\det(B) > 0$.

The trace of the matrix is

$$\mathrm{Tr}(B) = -\binom{n-1}{k-1}\phi_k x_k - \binom{n-1}{k}\phi_k x_k - \binom{n-1}{k-1}(1-\rho)\phi_k x_k - \nu\,. \qquad \text{(S37)}$$

Therefore, $\mathrm{Tr}(B) < 0$.

The eigenvalues of the diagonal sub-matrix D_1 are its diagonal terms: for all $i = 1, \dots, n$ such that $i \neq k$,

$$\lambda_i = \frac{\phi_i}{\phi_k} - 1\,. \qquad \text{(S38)}$$

Hence, since $\det(B) > 0$ and $\mathrm{Tr}(B) < 0$, the $(0, \dots, 0, \bar{x}_k, 0, \dots, 0, \bar{m})$ equilibrium is locally asymptotically stable if and only if $\phi_k > \phi_i$ for all $i = 1, \dots, n$ such that $i \neq k$.

S3.2.3 The $(0, \dots, 0, \bar{x}_n, 0)$ equilibrium

We consider the stability of the equilibrium $(0, \dots, 0, \bar{x}_n, 0)$ when it is positive, i.e. $\bar{x}_k > 0$. At the $(0, \dots, 0, \bar{x}_n, 0)$ equilibrium, we have $\frac{1}{n} - x_n = \frac{1}{\phi_n}$ (see Eq. S26). The jacobian matrix of size $(n+1) \times (n+1)$ evaluated around the $(0, \dots, 0, \bar{x}_n, 0)$ equilibrium is

$$J = \left(\begin{array}{ccccc|cc}
\frac{\phi_1}{\phi_n} - 1 & 0 & \dots & \dots & 0 & 0 & 0 \\
0 & \ddots & \ddots & & \vdots & \vdots & \vdots \\
\vdots & \ddots & \ddots & \ddots & \vdots & \vdots & \vdots \\
\vdots & & \ddots & \ddots & 0 & \vdots & \vdots \\
0 & \dots & \dots & 0 & \frac{\phi_{n-1}}{\phi_n} - 1 & 0 & 0 \\
\hline
-\binom{n-1}{0}\phi_n x_n & \dots & \dots & \dots & -\binom{n-1}{n-2}\phi_n x_n & \phi_n(\frac{1}{n} - 2x_n) - 1 & -\phi_n x_n \rho \\
\binom{n-1}{1}\frac{\phi_1}{\phi_n} & \dots & \dots & \dots & \binom{n-1}{n-1}\frac{\phi_n}{\phi_n} & 0 & -(1-\rho)\phi_n x_n - \nu
\end{array}\right).$$

The J matrix has the following form

$$J = \begin{pmatrix} D_2 & 0 \\ * & T \end{pmatrix},$$

where D_2 is a $(n-1) \times (n-1)$ diagonal matrix, $*$ is a $2 \times (n-1)$ matrix, and T is a 2×2 triangular matrix. Therefore, J is a block triangular matrix, and its eigenvalues

are the eigenvalues of D_2 and T, i.e. the diagonal terms.

Therefore, the eigenvalues are: for all $i = 1, \ldots, n-1$,

$$
\begin{aligned}
\lambda_i &= \frac{\phi_i}{\phi_n} - 1\,, \\
\lambda_n &= \phi_n \left(\frac{1}{n} - 2x_n \right) - 1\,, \\
\lambda_{n+1} &= -(1-\rho)\phi_n x_n - \nu < 0\,.
\end{aligned}
$$

Since $X = \frac{1}{n} - x_n$ (see Eq. S8), and $X = \frac{1}{\phi_n}$ (see Eq. S13),

$$
\lambda_n = \phi_n \left(\frac{1}{\phi_n} - x_n \right) - 1 = -\phi_n x_n < 0\,. \tag{S39}
$$

Therefore, the $(0, \ldots, 0, \bar{x}_n, 0)$ equilibrium is locally asymptotically stable if and only if $\phi_n > \phi_i$ for all $i = 1, \ldots, n-1$.

S3.3 Equilibria synthesis

To sum up, the Disease-free equilibrium $(0, \ldots, 0)$ always exists, and with $\phi_k < n$ for all $k = 1, \ldots, n$, it is locally asymptotically stable, and equilibria of the type $(0, \ldots, 0, \bar{x}_k, 0, \ldots, \bar{m}_k)$ and $(0, \ldots, 0, \bar{x}_n, 0)$ do not exist (in the positive orthant).

For each k such that $\phi_k > n$ with $k < n$, there exists an equilibrium of the type $(0, \ldots, 0, \bar{x}_k, 0, \ldots, \bar{m}_k)$ in the positive orthant. If $\phi_k > \phi_i$ for all $i = 1, \ldots, n$, there exists a unique equilibrium $(0, \ldots, 0, \bar{x}_k, 0, \ldots, \bar{m})$ which is locally asymptotically stable; in addition, the Disease-free equilibrium is unstable and the equilibrium $(0, \ldots, 0, \bar{x}_n, 0)$ does not exist (in the positive orthant).

Finally, if $\phi_n > n$, there exists one equilibrium of the type $(0, \ldots, 0, \bar{x}_n, 0)$ in the positive orthant. If $\phi_n > \phi_k$ for all $k = 1, \ldots, n-1$, the equilibrium $(0, \ldots, 0, \bar{x}_n, 0)$ is locally asymptotically stable, the Disease-free equilibrium is unstable, and the $n-1$ potential equilibria of the type $(0, \ldots, 0, \bar{x}_k, 0, \ldots, \bar{m}_k)$ do not exist (in the positive orthant).

S4 Warning about cases in which $1/c$ is an integer

Our model shows a competitive exclusion principle based on maximizing fitness. More specifically, a single equilibrium is asymptotically stable, the one containing the $\bar{x}_k$ that maximizes $\phi_k = kR(1-c)^k$. However, a non-generic case appears in the parameter situations raised in Theorem 1 where two virulence complexities coexist at equilibrium, and when this equilibrium corresponds to fitness maximization. In this case, there exists a k such that:

$$(k-1)R(1-c)^{k-1} = kR(1-c)^k\,, \tag{S40}$$

with $(k-1)$ and k on both sides of the maximum of the curve of Figure 1 in the main text. The above equation is equivalent to

$$\frac{k-1}{k} = \frac{(1-c)^k}{(1-c)^{k-1}}\,, \tag{S41}$$

yielding

$$c = \frac{1}{k}\,. \tag{S42}$$

This implies that two virulence complexities can have the same fitness. In this special case $\left(c = \frac{1}{k}\right)$, both virulence complexities $k-1$ and k can coexist (Fig. S4). However, this is a biologically non-generic case that is not studied further.

More specifically, Figure S4 shows infection dynamics with $n = 2$, for a non-generic case in which $c = \frac{1}{2}$. As in the previous generic case in which $c = 0.49$ (Fig. S2), the trajectories corresponding to the same virulence complexity converge from arbitrary initial conditions up to becoming identical (Fig. S4A), which allows us to switch from the full to the reduced model (Fig. S4B). At equilibrium, both virulence complexities are maintained and there is no competitive exclusion as is the case when $c \neq 0.5$ (Fig. S2). However, trajectories may not always converge, as shown in Figure S5 for $n = 3$. Technically, this observation challenges the symmetry assumption we made to justify the reduced model. Nevertheless, extensive simulations indicate that such a behaviour is the exception rather than the rule: it only happened in non-generic cases $\left(c = \frac{1}{k}\right)$ that are biologically irrelevant.

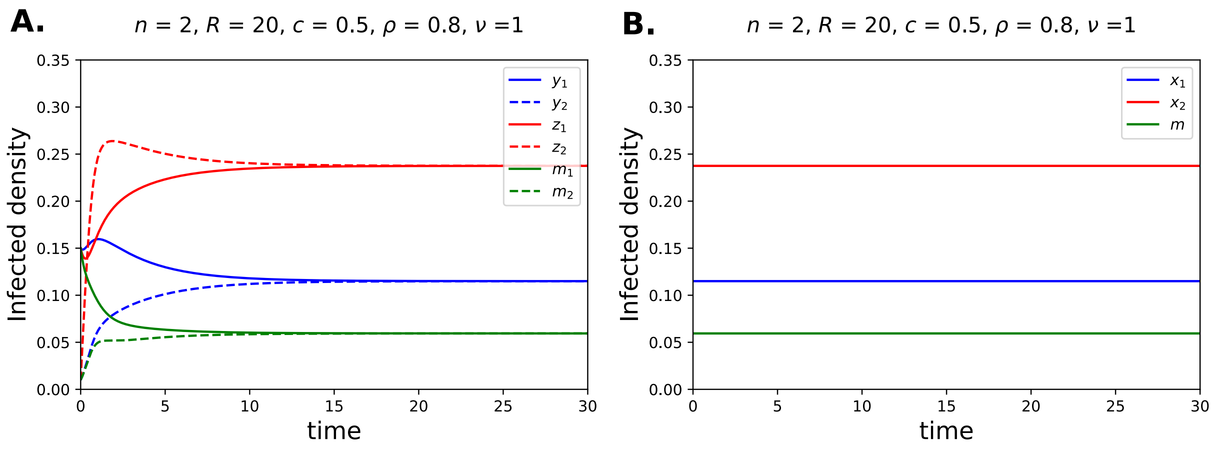

Figure S4: Infection dynamics over time for a model with $n = 2$ varieties for a non-generic case, since $\frac{1}{c}$ is an integer. The legend is the same as in Fig. S2. However, there is no competitive exclusion: virulence complexities 1 and 2 coexist in the long run.

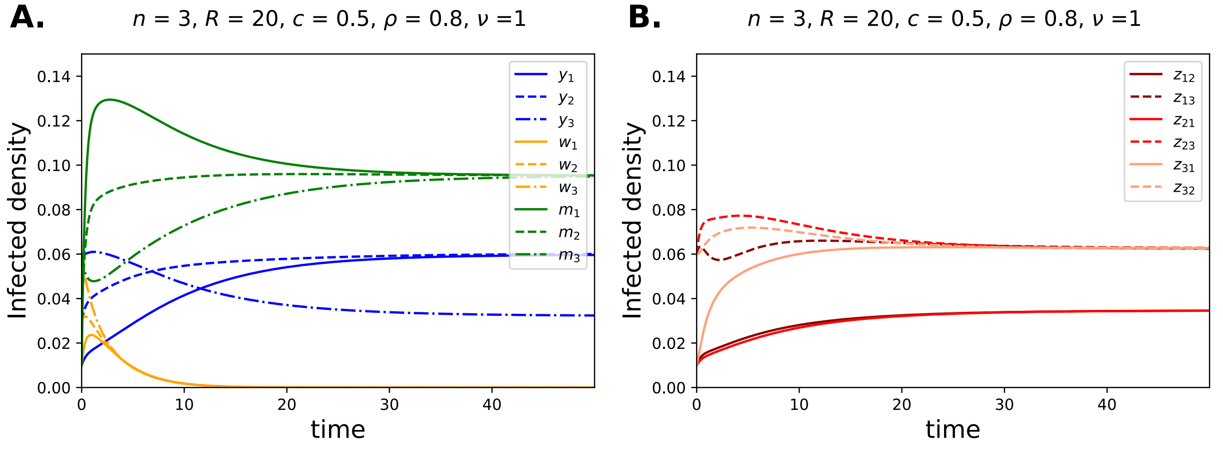

Figure S5: Infection dynamics over time for a model with $n = 3$ varieties for a non-generic case, since $\frac{1}{c}$ is an integer. The legend is the same as in Fig. S3. In this non-generic case, the trajectories corresponding to the same virulence complexity (e.g. the black curves y_i, $i = 1, 2, 3$), do not converge. Therefore, the full model cannot be reduced. However, this special case is biologically irrelevant.

S5 Prevalence of the disease

The prevalence of the disease at time t is defined as:

$$\mathcal{P}(t) = n \underbrace{\sum_{k=1}^{n} \binom{n-1}{k-1} x_k(t)}_{A} . \tag{S43}$$

As a reminder, the term A corresponds to the total density of infected hosts for each of the n varieties. This is the sum, over all possible virulence complexities, of the number of pathogen genotypes of complexity k able to infect a focal variety, $\binom{n-1}{k-1}$, multiplied by their individual density on the focal variety x_k.

S5.1 Prevalence at equilibrium

At endemic equilibrium, there is a unique virulence complexity k, which is the one maximizing the fitness function $\phi_k = kR(1-c)^k$, i.e.

$$k^* = \arg\max_{k=1,\ldots,n} \phi_k = \min\left(n, \arg\max_{k\in\mathbb{N}^*} \phi_k\right),$$

since n (the number of varieties in the mixture) is the maximum feasible virulence complexity, and ϕ_k is a unimodal function of k.

Let $\tilde{k} \in [1, n]$ be such that

$$\frac{\partial \phi_{\tilde{k}}}{\partial k} = 0\,.$$

This yields

$$\tilde{k} = -\frac{1}{\log(1-c)}\,,$$

which shows that the optimal virulence complexity does not depend on R (as can be seen immediately from the expression of ϕ_k). We have

$$\phi_{\tilde{k}} = -\frac{R}{e\log(1-c)}\,, \tag{S44}$$

where e is Euler's number.

Two cases can occur:

- If $k^* = n$, the selected equilibrium is such that $(0,\ldots,0,\bar{x}_n,0)$, with $\bar{x}_n > 0$. This means that there are no primed hosts at equilibrium. The density of uninfected and unprimed hosts for a focal variety (Eq. S8) simplifies as

$$X = \frac{1}{n} - \bar{x}_n = \frac{1}{\phi_n}\,,$$

after Eq. (S13). Therefore, the density of hosts of the focal variety that are infected is:

$$\bar{x}_n = \frac{1}{n} - \frac{1}{\phi_n}\,,$$

Hence the total equilibrium prevalence (for all varieties) is:

$$\bar{\mathcal{P}} = n\bar{x}_n = 1 - \frac{n}{\phi_n} = 1 - \frac{1}{R(1-c)^n}\,.$$

- Otherwise, if $k^* < n$, the selected equilibrium is such that $(0,...,0,\bar{x}_k^*,0,...,\bar{m}_{k^*})$, where $\bar{m}_{k^*}$ is the density of hosts of the focal variety that are primed. Combining Eq. (S8) and Eq. (S13), we have:

$$\frac{1}{n} - \bar{m}_{k^*} - \binom{n-1}{k^*-1}\bar{x}_{k^*} + (1-\rho)\bar{m}_{k^*} = \frac{1}{\phi_{k^*}},$$

which is rearranged to obtain the density of hosts of the focal variety that are infected:

$$\binom{n-1}{k^*-1}\bar{x}_k^* = \frac{1}{n} - \rho\bar{m}_{k^*} - \frac{1}{\phi_{k^*}},$$

Therefore, the total prevalence (for all varieties) is:

$$\bar{\mathcal{P}} = n\binom{n-1}{k^*-1}\bar{x}_{k^*} = 1 - n\left(\rho\bar{m}_{k^*} + \frac{1}{\phi_{k^*}}\right).$$

In the priming-free case ($\rho = 0$), the total prevalence simplifies as

$$\bar{\mathcal{P}} = 1 - \frac{n}{\phi_{k^*}},$$

meaning that the prevalence decreases linearly with n. Approximating ϕ_{k^*} with $\phi_{\tilde{k}}$ (Eq. S44), we get

$$\bar{\mathcal{P}} \approx 1 - \frac{n}{\phi_{\tilde{k}}} = 1 + n\frac{e\log(1-c)}{R}.$$

Therefore, the critical number of varieties required to eradicate the disease (i.e. such that $\bar{\mathcal{P}} = 0$) is

$$n_c = \frac{R}{-e\log(1-c)}.$$

This quantity increases linearly with R and decreases much faster than linearly with c (Figure S6).

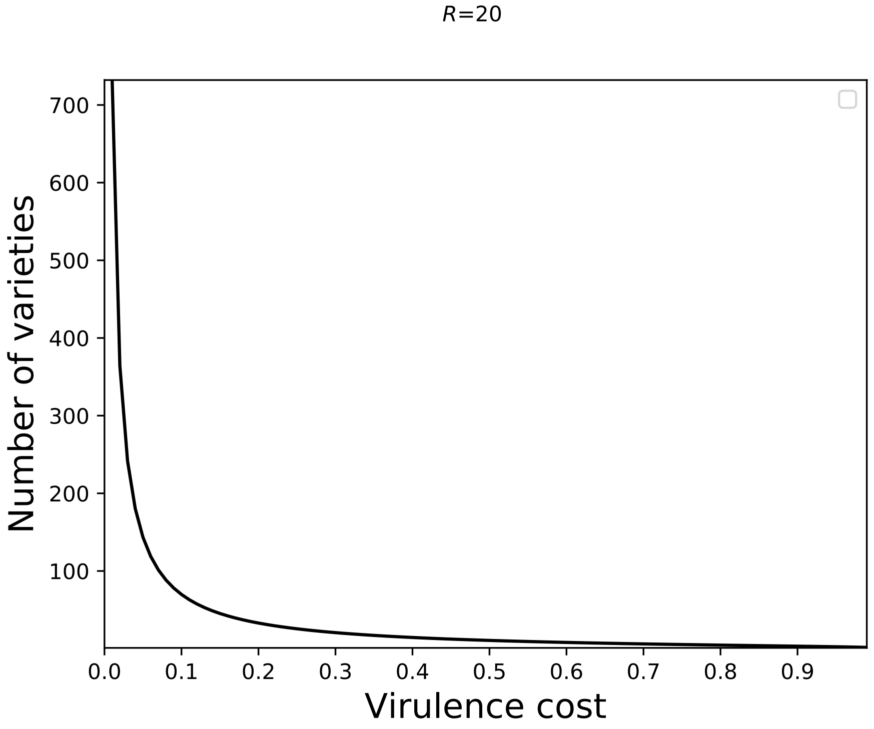

Figure S6: Critical number n_c of varieties to be used in the mixture to eradicate the disease depending on c (virulence cost), for $R = 20$ (transmission rate).

S6 Area Under Disease Progress Curve (AUDPC)

We define the area under the disease progress curve (AUDPC) as follows:

$$AUDPC(t) = \int_0^t \mathcal{P}(\tau) d\tau \,. \tag{S45}$$

The AUDPC is a standard metric to summarize the epidemic size at time t because it takes into account the speed at which the epidemic spread from time 0 to time t.

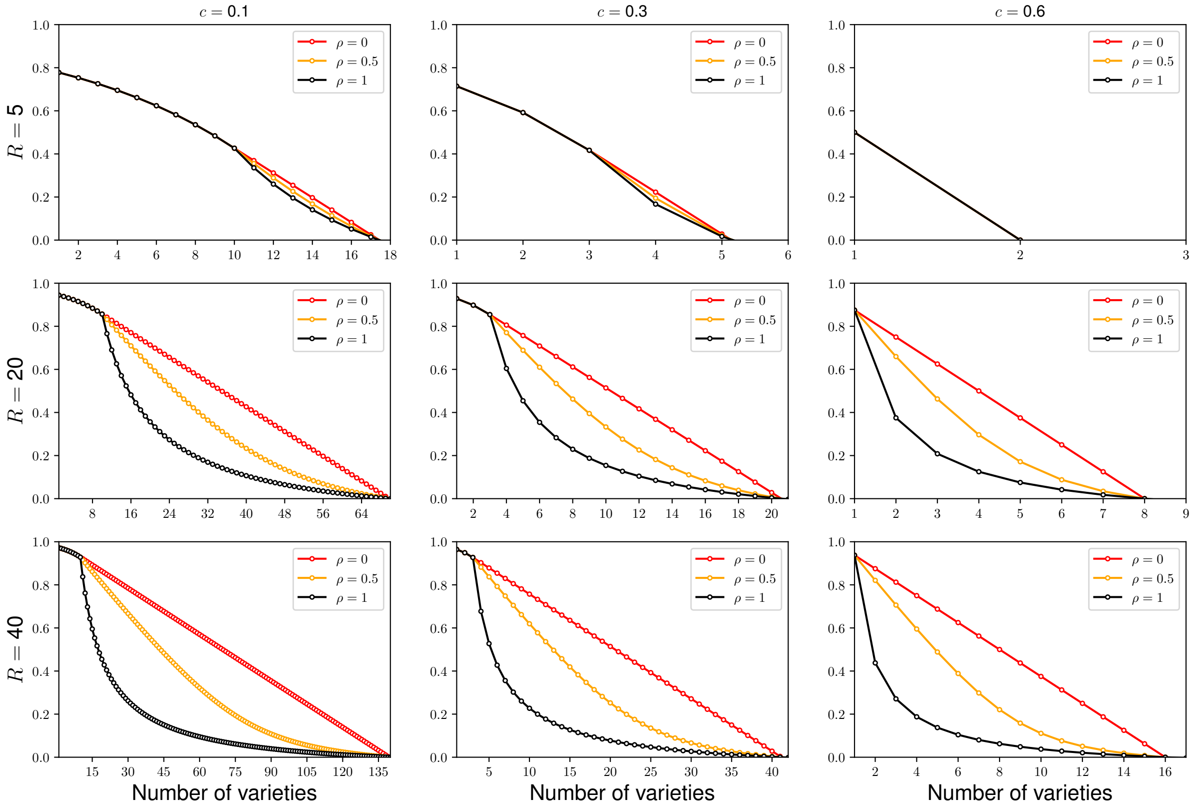

Figure S7: Prevalence of the disease at equilibrium $\bar{\mathcal{P}}$ as a function of the number of varieties n for a set of transmission rates R (rows), virulence costs c (columns), and priming efficiencies ρ (legends), with re-scaled removal rate $\nu = 1$.

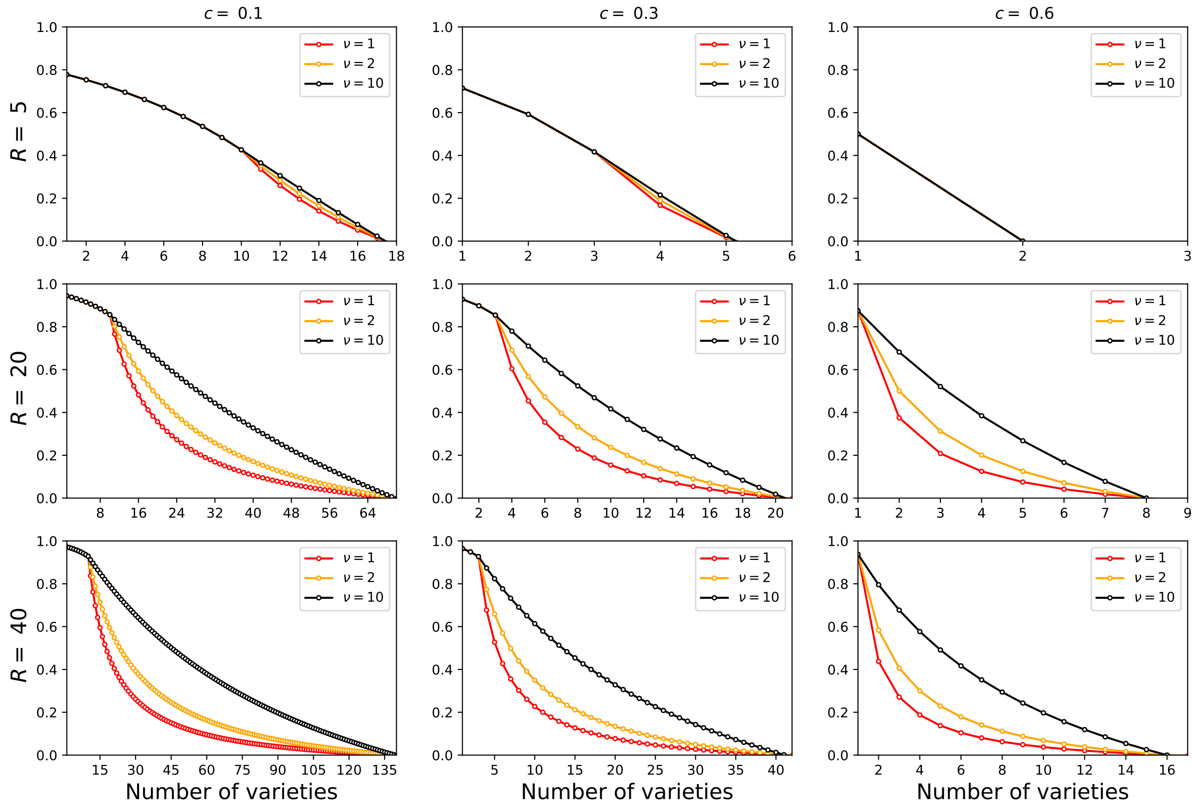

Figure S8: Prevalence of the disease at equilibrium $\bar{\mathcal{P}}$ as a function of the number of varieties n for a set of transmission rates R (rows), virulence costs c (columns), and re-scaled removal rates ν (legends), with priming efficiency $\rho = 1$.

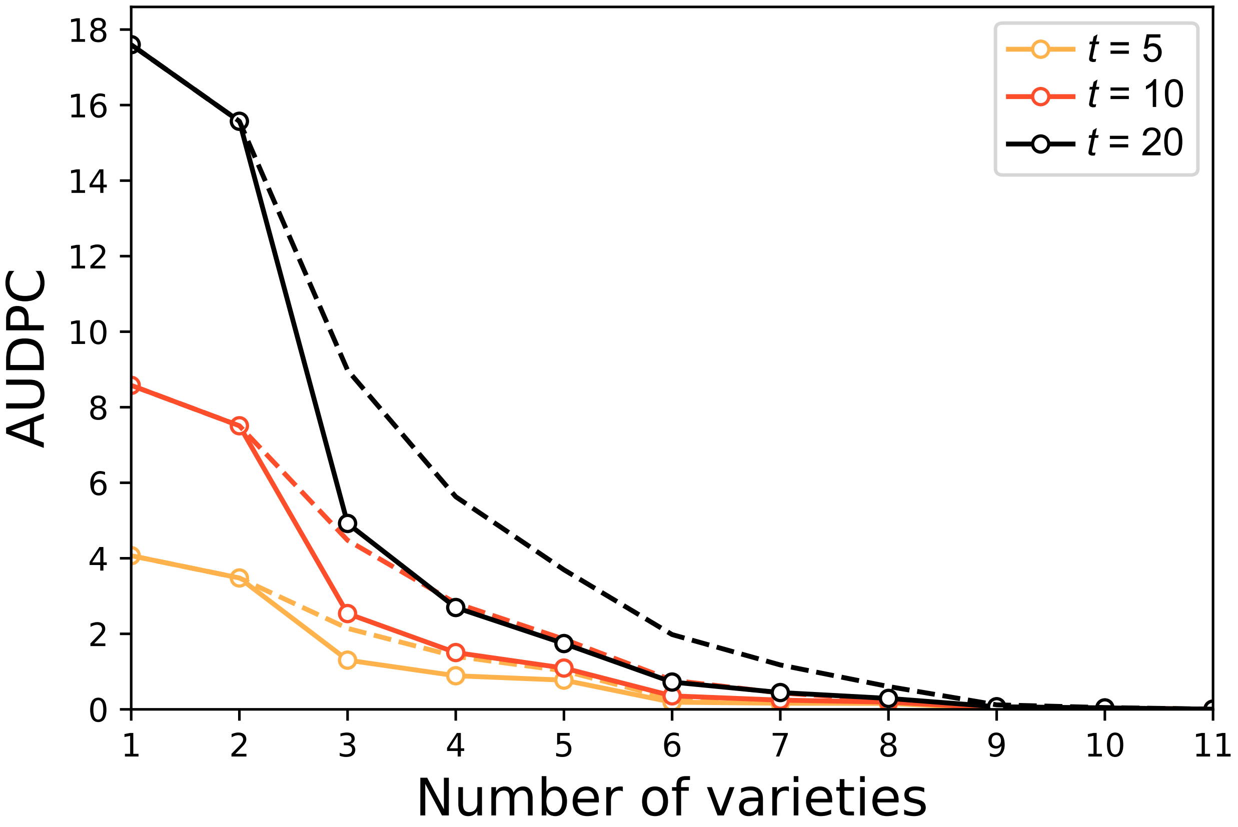

Figure S9: The Area Under the Disease Progress Curve (AUDPC) over time t, and as a function of the number of lines n in the mixture. The parameters are $R = 20$ (transmission rate), $c = 0.49$ (virulence cost) and $\nu = 1$ (re-scaled removal rate). The full and dotted line correspond to $\rho = 1$ (full priming) and $\rho = 0$ (no priming). The initial conditions were arbitrarily set as: $m(0) = x_k(0) = 0.01/(n(1 + 2^{n-1}))$ for all $k = 1, \ldots, n$.
